# PSB33 sustains photosystem II D1 protein under fluctuating light conditions

**DOI:** 10.1093/jxb/erx218

**Published:** 2017-07-26

**Authors:** Rikard Fristedt, Andrea Trotta, Marjaana Suorsa, Anders K Nilsson, Roberta Croce, Eva-Mari Aro, Björn Lundin

**Affiliations:** 1 Department of Physics and Astronomy, Faculty of Sciences, VU University Amsterdam, De Boelelaan, Amsterdam, The Netherlands; 2 Department of Biochemistry, Molecular Plant Biology, University of Turku, Finland; 3 Department of Biological and Environmental Sciences, University of Gothenburg, Gothenburg, Sweden

**Keywords:** Arabidopsis, chloroplast, fluctuating lights, photosynthesis, PSB33, PSII, quality control of PSII, state transition

## Abstract

On Earth, solar irradiance varies as the sun rises and sets over the horizon, and sunlight is thus in constant fluctuation, following a slow dark–low–high–low–dark curve. Optimal plant growth and development are dependent on the capacity of plants to acclimate and regulate photosynthesis in response to these changes of light. Little is known of regulative processes for photosynthesis during nocturnal events. The nucleus-encoded plant lineage-specific protein PSB33 has been described as stabilizing the photosystem II complex, especially under light stress conditions, and plants lacking PSB33 have a dysfunctional state transition. To clarify the localization and function of this protein, we used phenomic, biochemical and proteomics approaches in the model plant Arabidopsis. We report that PSB33 is predominantly located in non-appressed thylakoid regions and dynamically associates with a thylakoid protein complex in a light-dependent manner. Moreover, plants lacking PSB33 show an accelerated D1 protein degradation in nocturnal periods, and show severely stunted growth when challenged with fluctuating light. We further show that the function of PSB33 precedes the STN7 kinase to regulate or balance the excitation energy of photosystems I and II in fluctuating light conditions.

## Teaser

The chloroplast protein PSB33 is primarily located in non-appressed thylakoid regions, and is vital for growth of plants in fluctuating light through regulation of PSII quality control.

## Introduction

The natural environmental conditions are characterized by frequent predictable (diurnal changes) and unpredictable (clouds, wind) fluctuations in light quality and quantity. Thus, to grow and reproduce, plants need to have regulatory flexibility in their photosynthetic machinery and an active repair system to avoid photoinhibition (Fristedt *et al.*, 2009*a*; Demmig-Adams *et al.*, 2012; Croce and van Amerongen, 2014). The importance of functional flexibility of the light-harvesting complex (LHCII) antenna in balancing the excitation efficiencies of potosystems (PS) I and II has been studied in great detail (Wientjes *et al.*, 2013; Pietrzykowska *et al.*, 2014; Grieco *et al.*, 2015). It has been shown that the reversible phosphorylation of LHCII triggers the equal allocation of light energy to PSI and PSII, a process known as state transition. The kinase (STN7) and phosphatase (PPH1/TAP38, hereafter TAP38) proteins responsible for this process have been characterized (Bellafiore *et al.*, 2005; Pribil *et al.*, 2010*b*; Shapiguzov *et al.*, 2010). Since its discovery, the regulation of STN7 has been extensively studied (Pesaresi *et al.*, 2009; Pribil *et al.*, 2010*a*). The phosphorylation of the PSII core proteins in Arabidopsis depends on the redox-activated protein kinase STN8 and controls the overall light-induced flexibility of the thylakoid membrane, which in turn regulates the PSII core D1 protein turnover during PSII quality control (Tikkanen *et al.*, 2008; Fristedt *et al.*, 2009*b*; Tikkanen and Aro, 2012). The processes involved in the quality control of PSII are tightly regulated and involve several auxiliary proteins (Järvi *et al.*, 2015). More specifically, the core proteins of damaged PSII are phosphorylated by STN8, and PSII is partially disassembled, followed by the migration of damaged PSII subcomplexes to the stroma lamellae, where insertion of the new D1 protein and reassembly of the PSII core complex take place. Finally, PSII is dimerized, and the supercomplex is again formed in the grana regions (Baena-González and Aro, 2002). Although the individual steps in PSII quality control have been studied in great detail, this process is still poorly understood.

The chloroplast-located protein PSB33 has a crucial role in maintaining the stability of PSII and regulating photosynthesis (Fristedt *et al.*, 2015), but the mechanisms surrounding its function have remained unclear. *psb33* belongs to a group of genes called the GreenCut that have recently gained a lot of attention and are characterized by the feature that they are only found in organisms performing oxygenic photosynthesis (Merchant *et al.*, 2007; Fristedt, 2017). It was recently shown that the amount of PSB33 follows a diurnal cycle (Wang *et al.*, 2016*a*) and that the protein possibly interacts with EXECUTER1 (EX1) at the grana margins (Wang *et al.*, 2016*a*). Interestingly, Cruz *et al.*, (2016) further showed that under light conditions mimicking natural environments (fluctuating light), *psb33* plants display a phenotype described as ‘extremely patchy’, where the fluorescence response appears to be sporadic in leaves when monitoring photosynthetic performance over the whole plant under highly fluctuating growth light conditions for 5 days.

In this paper, specific emphasis was placed on the biochemical and proteomic analysis of the *psb33* mutant to investigate the involvement of the PSB33 protein in the regulation of photosynthesis under changing light conditions. We show that PSB33 is primarily localized to non-appressed thylakoid regions, and we present evidence that it is crucial to maintaining PSII under fluctuating light conditions.

## Methods

### Growth conditions and plant material

Wild-type *Arabidopsis thaliana* accession Columbia-0 (Col-0) plants and the knock-out mutant lines *psb33-3* (Fristedt *et al.*, 2015) and *stn7* (Bellafiore *et al.*, 2005) were used in this study. Plants were cultivated in climate-controlled chambers in the short day condition (8 h day/16 h night, 22 °C day/18 °C night) at a photon flux of 120 μmol m^−2^ s^−1^, provided with fluorescence lamps, and a relative humidity of 60%, unless stated otherwise. The conditions used for the growth phenotype under fluctuating light were those described in Grieco *et al.* (2012). The light intensities referred to as low light (LL), growth light (GL), and high light (HL) were 50, 130, and 500 μmol photons m^−2^ s^−1^, respectively, provided with a metal halide lamp. The plants used in the experiments were 5–6 weeks old.

### Subfractionation of thylakoid membranes

Thylakoids were diluted to 1 mg ml^−1^ chlorophyll by adding resuspension buffer (25 mM Tricine-NaOH, pH 7.8, 150 mM sorbitol, 10 mM NaCl, and 5 mM MgCl_2_). Membranes (at 0.5 mg ml^−1^ chlorophyll concentration) were solubilized for 15 min on ice in the same solution in the presence of 1% digitonin or 0.1% dodecyl maltoside. The reaction was stopped by adding a 10-fold volume of ice-cold resuspension buffer. After centrifugation at 1000 *g* for 3 min at 4 °C, supernatant was collected, and grana lamellae were collected by centrifugation at 10 000 *g* for 30 min at 4 °C. The supernatant was centrifuged at 40 000 *g* for 30 min at 4 °C to collect the grana margins. Finally, to pellet stroma lamellae-enriched membranes, the supernatant was centrifuged at 145 000 *g* for 1 h at 4 °C.

### SDS-PAGE and immunodetection

Total leaf protein extracts were prepared by grinding tissue in liquid nitrogen following the addition of extraction buffer (100 mM Tris–HCl pH 8.0, 25 mM EDTA pH 8.0, 0.25 M NaCl, 0.75% SDS, 10 mM NaF, 1 mM DTT, protease inhibitor) and heat denaturation (68 °C 10 min). The relative protein concentration was determined based on chlorophyll content using the Porra method (Porra *et al.*, 1989). Protein extracts diluted in 3× loading buffer (Tris–HCl pH 8.0, 40% glycerol, 0.1% SDS, 10 mM NaF, 1 mM DTT) were separated on 5%/14% acrylamide stacking/separation gel and blotted onto nitrocellulose membranes (Amersham, GE Healthcare, www.gehealthcare.com) through semi-wet transfer. Membranes were blocked with 1% bovine serum albumin (BSA) and were then incubated with antibodies for Lhcb1, Lhcb2 or their respective phosphorylated forms (all from Agrisera, Vännäs, Sweden, http://www.agrisera.com,). Membranes were then incubated with secondary anti-rabbit antibody conjugated with horseradish peroxidase (Agrisera) and were detected using ELC detection on an LAS-3000 imager (Fuji).

### Large pore blue native gel electrophoresis

For native PAGE, thylakoids were solubilized with 1% digitonin as previously described (Suorsa *et al.*, 2015), separated with large pore blue native (lpBN)-PAGE as previously described (Järvi *et al.*, 2011), and either stained with Coomassie brilliant blue or electroblotted to a polyvinylidene difluoride (PVDF) membrane. For one-dimensional SDS-PAGE, thylakoids were solubilized, separated by gel electrophoresis with 15% (w/v) polyacrylamide and 6 M urea and subsequently electroblotted. For 2D gels, the second dimension was run for 3 h at 200 V for an additional ‘3D’ dimension, and the band corresponding to PSII monomer/cytochrome *b*_6_*f* (Cyt *b*_6_*f*) was immediately placed on top of a second BN gel with a gradient of 7.5–9.5%.

The antibodies used for immunoblotting were Lhcb1, Lhcb2, P-Lhcb1, P-Lhcb2, CP47, PSB33, STN7 (Agrisera; catalogue numbers AS09 522, AS01 003, AS13 2704, AS13 2705, AS04 038, AS12 1852 and AS10 1611) and TAP38. The TAP38 antibody was a kind gift from Prof. Roberto Barbato. Immunodetection was performed according to standard procedures, with horseradish peroxidase-linked secondary antibody and enhanced chemiluminescence reagents (Amersham, GE Healthcare) used for detection.

### Quantum yield of PSII and PSI measurements

The quantum yield of PSII (YII) and PSI (YI) was measured with a DUAL-PAM 100 measuring system (Walz, Effeltrich, Germany). Plants were dark adapted for 30 min before measuring with intervals every minute at given light intensity. YII and YI were calculated as (*F*_m_′−*F*)/*F*_m_′ and (*P*_m_′−*P*)/*P*_m_, respectively.

### Selected reaction monitoring analysis

Selected reaction monitoring (SRM) analysis of wild-type and *psb33* plants exposed to the previously described light regime of dark, LL1, HL, and LL2 was performed as previously described in (Trotta *et al.*, 2016). In total, three biological and two technical replicates were used for wild-type plants, and two biological replicates were used for *psb33.* The proteotypic peptides for each protein were chosen using the platform Arabidopsis Proteotypic Predictor (APP) (http://www.plantenergy.uwa.edu.au/APP/) (Taylor *et al.*, 2014). The list of peptides and related transitions used for relative quantification is provided in Supplementary Table S1 at *JXB* online. The full set of biological and technical replicates of the refined SRM dataset has be deposited in Panorama public and can be accessed via the following link: https://panoramaweb.org/labkey/PSB33_thylakoids_ratios.url

The phosphorylation stoichiometry for TVAKPK (p-Thr lhcb1) has been calculated as the percentage of the phosphorylated form with respect to the sum of the phosphorylated and non-phosphorylated form, using the formulas described in (Trotta *et al.*, 2016). The phosphorylation stoichiometry for ELEVIHS[+80]RWAMLGALGC[+57]VFPELLAR (p-Ser lhcb1) and ELEVIHS[+80]RWAMLGALGC[+57]TFPEILSK (p-Ser lhcb2) has been estimated as percentage of the respective unique phosphorylated peptide for lhcb1 or for lhcb2, with respect to the sum of all the non-phosphorylated peptides containing the sequence ELEVIHS, assuming that the level of phosphorylation is similar for the two isoforms.

## Results

### Fluctuating light severely affects *psb33* plant growth

**Fig. 1.**
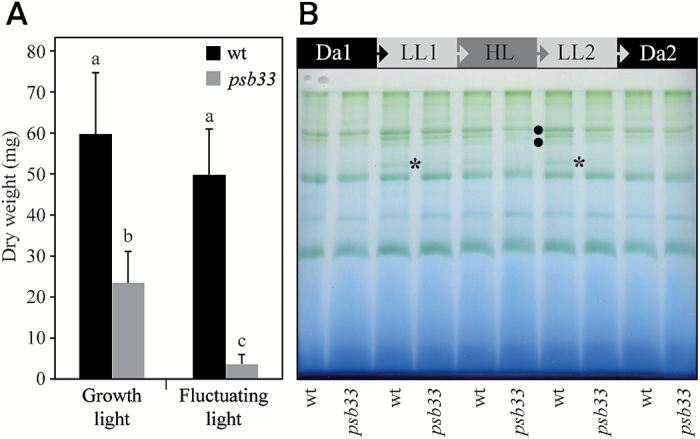
Arabidopsis *psb33* mutant is impaired in its antenna size adjustment in response to fluctuating light. (A) Dry weight of aerial plant tissue from wild-type (wt) and *psb33* mutant plants grown for 5 weeks under constant or fluctuating light conditions. Averages and standard deviations (*n*=9–14) are shown. Letters above bars indicate statistically significant differences determined by one-way ANOVA with Tukey’s *post hoc* test (*P*<0.005). (B) Dynamics of thylakoid membrane protein complexes in response to varying light conditions in wt and *psb33* plants. Thylakoids were isolated from plants acclimated to 16 h of dark (Da1), followed by 2 h of low light (LL1), 2 h of high light (HL), 2 h of low light 2 (LL2), and finally 2 h of dark (Da2). Thylakoids were solubilized with digitonin and then centrifuged and the supernatant was separated by lpBN gel. The state transition-specific complex (PSI–LHCI–LHCII) is indicated by an asterisk, and the higher molecular mass complexes showing differences between wt and *psb33* are indicated by bullets. (This figure is available in color at *JXB* online.)

We recently characterized Arabidopsis *psb33* mutants as being defective in state transitions, as determined by chlorophyll *a* fluorescence measurements (Fristedt *et al.*, 2015). To address whether *psb33* plants exhibit a visible phenotype under fluctuating light conditions, we exposed *psb33* and wild-type plants to a series of low to high light intensity growth conditions (8-h photoperiod consisting of 5 min of 60 photon flux density (PFD; μmol photons m^−2^ s^−1^), and 1 min of 600 PFD), which has negatively affected the growth of other state transition mutants, including *stn7* (Grieco *et al.*, 2012) and *psal* (Grieco *et al.*, 2012). In comparison to *stn7* and *psal*, *psb33* growth was severely affected under these condition; the rosette dry mass of *psb33* was only 7% of the wild-type mass, compared with 39% under constant growth light (Fig. 1A).

### Formation of the state transition complex (PSI–LHCII–LHCI) upon changing light conditions

The apparent loss of artificially induced state transitions in *psb33* (Fristedt *et al.*, 2015) led us to further investigate the formation of the PSI–LHCII–LHCI complex (Kouřil *et al.*, 2005) during natural mimicking changing light conditions. To this end, leaves were harvested from plants before the onset of light (after 16 h of darkness, Da1), followed by treatment first for 2 h with low light (LL1) (50 PFD), then 2 h with high light (HL) (800 PFD), again with 2 h low light (LL2) (50 PFD), and finally for 2 h in darkness (Da2). This light cycle treatment emulates natural daylight conditions and triggers reorganization of the moveable LHCII antenna (Suorsa *et al.*, 2015). To prevent the dissociation of the protein complexes, thylakoids were isolated rapidly from fresh leaves after each time point. Isolated thylakoids were treated with digitonin to solubilize the non-appressed thylakoid domains, which then were subjected to the separation of protein complexes by large pore blue native (lpBN) gel electrophoresis (Fig. 1B; Järvi *et al.*, 2011). In wild-type plants, the state transition-specific complex was barely detectable under darkness; however, the shift of plants to LL1 induced a strong accumulation of the complex (Fig. 1B asterisk). The complex was again not detectable when the plants shifted to HL, while the subsequent shift to LL2 again showed a substantial increase in the amount of the PSI–LHCI–LHCII complex comparable to the Da1–LL1 shift. Finally, when the wild-type plants shifted to Da2 again, the PSI–LHCI–LHCII complex disappeared, according to the known reversible assembly and disassembly dynamics described in (Suorsa *et al.*, 2015; Trotta *et al.*, 2016). The *psb33* mutant, in turn, showed only negligible amounts of the PSI–LHCI–LHCII complex upon the Da1–LL1 shift and no traceable amounts of this complex upon shifts to HL or LL2.

### PSB33 is mainly localized to the non-appressed stroma lamellae region of the thylakoid membrane

**Fig. 2.**
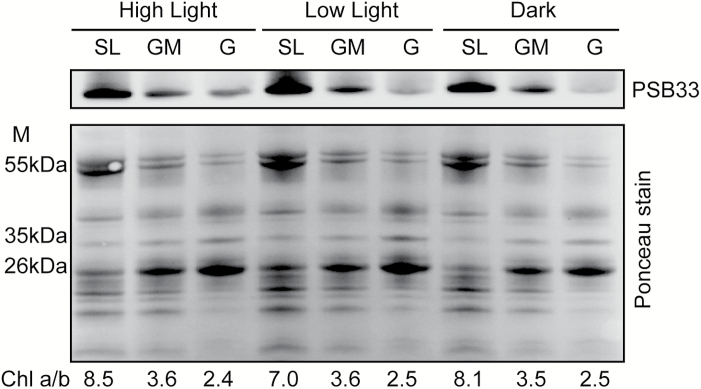
PSB33 protein is mainly located in non-appressed thylakoids. Digitonin-solubilized thylakoid fractions (stroma lamellae (SL), grana margins (GM), and grana (G)) from wild-type plants acclimated to dark, low light (50 PFD), and high light (800 PFD) for 2 h were separated on an SDS gel, transferred to a nitrocellulose membrane and subsequently immunodecorated with an antibody against PSB33. A total protein stain (Ponceau) of the membrane shows that the same amount of protein was loaded and a clear protein-specific pattern of fractionation.

We previously localized PSB33 to the vicinity of LHCII and the PSII reaction center protein CP43 (Fristedt *et al.*, 2015). As PSB33 affects the state transition complex PSI–LHCI–LHCII, it is highly conceivable that its function is not limited to static conditions. To this end, we next addressed the localization of PSB33 in differentially light-acclimated plants. Thylakoids from 2-h dark-, growth-light- and high-light-acclimated wild-type plants were fractionated into grana, grana margins and stroma-exposed thylakoid domains, followed by immunodetection with an antibody against PSB33 (Fristedt *et al.*, 2015). In all conditions, the PSB33 protein largely located to non-appressed thylakoid regions, and only a small portion of PSB33 was located to the grana (Fig. 2).

### PSB33 forms different complexes in non-appressed thylakoid membrane

**Fig. 3.**
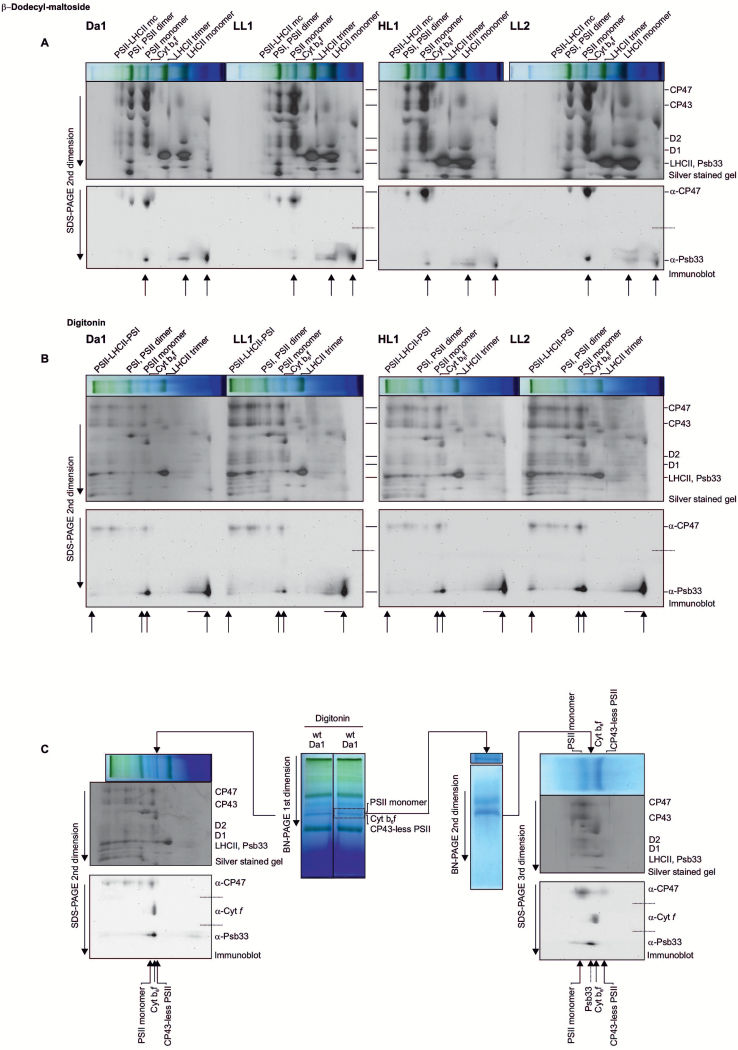
PSB33 thylakoid membrane association upon changes in light conditions. Localization of the main complexes containing PSB33 in appressed and non-appressed thylakoid domains by lpBN-PAGE followed by SDS-PAGE in the second dimension, partial blotting of the gels and overlap of the images obtained by silver-staining of the gels and further immunodetection with antibodies against CP47, Cyt *f*, and PSB33. The dashed line indicates where the PVDF membranes were separated into two or three parts and subsequently probed separately with the two or three antibodies indicated to avoid cross-reacting signals. Major thylakoid complexes are indicated on top, and the PSII core subunits are indicated on the side. The complexes containing PSB33 are indicated by arrows at the bottom. (A) β-Dodecyl-D-maltoside-solubilized thylakoid extracts corresponding to 6 μg of Chl from wild-type plants treated with 16 h of dark (Da1) followed by 2 h of low light (LL1), 2 h of high light (HL), and another 2 h of low light (LL2). Upper panel: lpBN strip and silver-stained SDS-PAGE second dimension. Lower panel: immunoblot from the same gel probed with CP47 and PSB33 antibodies. The last arrows at the right of each second dimension indicate free protein. (B) Digitonin-solubilized thylakoid extracts corresponding to 6 μg of Chl from the same samples as in (A). Upper panel: lpBN strip and silver-stained SDS-PAGE second dimension. Lower panel: immunoblot from the same gel probed with CP47 and PSB33 antibodies. The last arrows at the right of each second dimension indicate free protein. (C) A method for the separation and detailed resolution of comigrating complexes in consecutive PAGE steps. Two samples of digitonin-solubilized thylakoid extracts from dark-acclimated wild-type plants, corresponding to 8 μg of Chl, were separated into two lanes of the same lpBN (central part of the figure, marked as first dimension). One lane was used for the second dimension in SDS-PAGE, as in (B) (on the left). The molecular mass area corresponding to PSII monomer/Cyt *b*_6_*f* was excised from the second lane (dashed rectangle) and placed on top of a second BN-PAGE (7.5 to 10% gradient, indicated as the second dimension on the right) to further separate the protein complexes, which were finally separated into their subunit compositions by SDS-PAGE (indicated as third dimension, on the right) and partially blotted as described above to be probed with CP47, Cyt *f* and PSB33 antibodies. The separated complexes are indicating by arrows at the bottoms of both immunoblots. (This figure is available in color at *JXB* online.)

Next, we sought to determine whether PSB33 was associated with other complexes in the appressed and non-appressed regions of the thylakoid membrane and if these associations changed in the different light conditions described in Fig. 1. To this end, the thylakoids were solubilized, and the protein complexes were separated in lpBN gels (Fig. 3A, B, and Supplementary Fig. S1), followed by a second-dimension run with SDS-PAGE. The 2D gel was then partially immunoblotted and probed with antibodies against CP47 and PSB33, while the remaining proteins in the partially blotted gel were silver stained. The two images (gel and immunoblot) were overlapped to obtain an accurate localization of PSB33 in the 2D map (Fig. 3A, B). As shown in Fig. 3A, in the appressed thylakoid regions where only low amounts of PSB33 were found (Fig. 2), a well-resolved minor spot apparently comigrated with PSII monomers, while most of the protein was comigrating with LHCII monomers and as a free protein (Fig. 3A, indicated by arrows). Interestingly, the intensity of the highest molecular mass spot was influenced by the light treatment, showing a decrease in the shift Da1–LL1 (20% to 8%) (Fig. 3A). In the non-appressed thylakoid regions of dark-acclimated plants, PSB33 was present in several complexes (Fig. 3B, indicated by arrows). Two smaller spots were found close to Cyt *b*_6_*f* and PSII monomer, following the same light treatment-dependent trend observed for the minor spot in the appressed thylakoid region (Fig. 3B). To clarify whether the latter two spots were associated with the PSII and/or Cyt *b*_6_*f* complex, an additional separation step was performed for this molecular mass region of the lpBN gels, as previously described (Rahikainen *et al.*, 2017; Fig. 3C, in the center). Wild-type plant thylakoids from Da1, LL and HL were treated with digitonin and the solubilized non-appressed domains were fractionated in BN-PAGE gels and a subsequent second dimension with SDS-PAGE (Fig. 3C, to the left). In parallel, the molecular mass region comprising the PSII monomer/Cyt *b*_6_*f* bands (indicated by a dashed line rectangle in Fig. 3C, in the center) was excised from an identical BN lane and placed on top of a second BN-PAGE gel with a different acrylamide gradient. The obtained strip was finally placed on top of an SDS-PAGE gel, and the protein complexes separated into their subunits in a third dimension (Fig. 3C, to the right). In both cases, the gels were partially immunoblotted and probed with antibodies against CP47, Cyt *f* and PSB33. Interestingly, one of the two PSB33 spots migrated as a higher molecular mass complex than the PSII monomer; the other spot was between the PSII monomer and the Cyt *b*_6_*f* complex, suggesting that PSB33 likely interacts with a currently undisclosed complex in the non-appressed thylakoid region.

### Dynamic thylakoid protein phosphorylation is defective in *psb33* plants

**Fig. 4.**
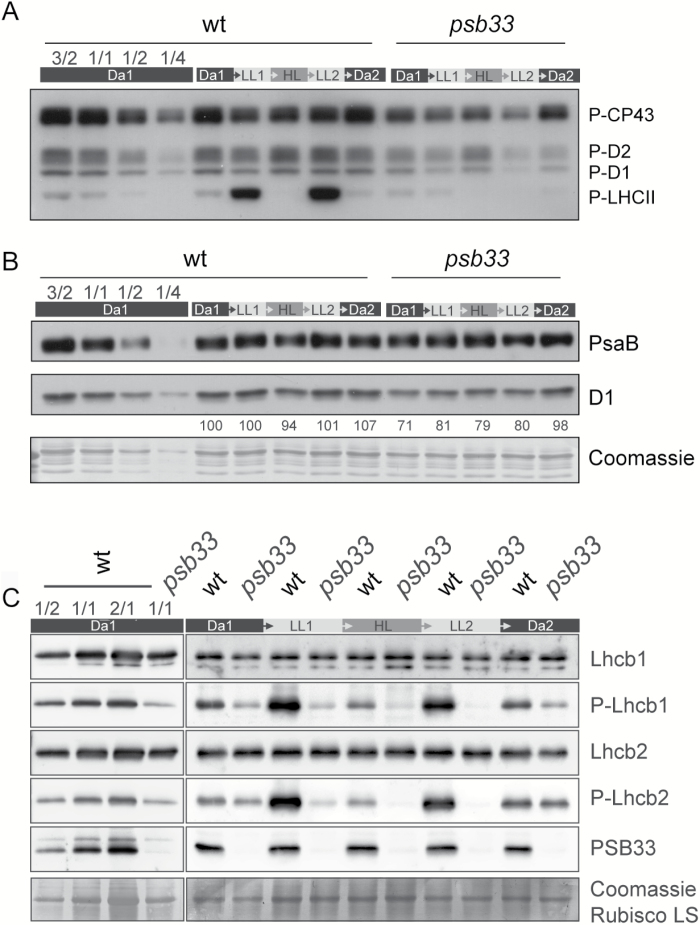
Reversible PSII-LHCII phosphorylation is defective in *psb33* plants in fluctuating light conditions. Dynamics of thylakoid membrane phosphoproteins in response to fluctuating light conditions in wt and *psb33*. Thylakoids were isolated from plants at the end of the five dark–light periods (dark (Da1), low light for 2 h (LL1), high light for 2 h (HL), low light for 2 h (LL2), and darkness overnight (Da2)). On the left, a dilution series of dark (Da1) acclimated wild-type thylakoids shows the linear range of the antibody. (A) Immunoblot analyses of the PSII core (P-CP43, P-D2 and P-D1) and LHCII (P-LHCII) phosphoproteins from whole thylakoids were performed after SDS-PAGE. (B) Immunoblot showing the amounts of PsaB (above) and D1 (below) in the same samples as (A). Quantitative D1 protein levels, which have been normalized to Da1 D1 levels, are represented as numeric values below the D1 immunoblot. A Coomassie-stained PVDF membrane is shown as a loading control. (C) Immunoblots with specific antibodies against Lhcb1, P-Lhcb1, Lhcb2, P-Lhcb2, and PSB33 of whole-leaf protein extracts from wild-type and *psb33* plants, highlighting the change in phosphorylation but not protein amounts of Lhcb1 and Lhcb2. A Coomassie-stained PVDF membrane is shown to demonstrate equal loading.

The dynamic phosphorylation status of thylakoid proteins regulates various photosynthetic processes, such as acclimation to low light (LHCII phosphorylation) and the PSII repair cycle (PSII core phosphorylation) (Tikkanen *et al.*, 2008; Fristedt *et al.*, 2009*b*; Rochaix *et al.*, 2012). To study these mechanisms in *psb33*, the level of phosphorylation of the major thylakoid phosphoproteins was assessed in Da1, LL1, HL, LL2, and Da2 samples with a specific phospho-threonine antibody. As shown in Fig. 4A, LHCII phosphorylation was induced by low light in wild-type plants, while the phosphorylation of the PSII core phosphoproteins CP43, D2, and D1 reached its maximum under high light, which at the same time dephosphorylated LHCII proteins (Tikkanen *et al.*, 2008). Importantly, the shifting of plants to low light again induced LHCII phosphorylation, as also shown in Trotta *et al.* (2016). Further we observed a slow dephosphorylation of PSII core proteins after high light exposure. This may act as a protective mechanism to avoid excess degradation of PSII that is active in the second low light period to repair damaged PSII (Kato and Sakamoto, 2014). In contrast, there was no induction of LHCII protein phosphorylation in LL1 for *psb33* plants, neither after the high-light treatment nor in the following low-light and dark conditions (Fig. 4A). The lower phosphorylation visible for PSII core proteins in *psb33* plants (Fig. 4A) prompted us to test whether the amounts or activity of PSI and PSII were affected in these plants. Notably, while PsaB showed unaltered levels between wild-type and mutant plants, the level of D1 protein was already low in *psb33* before application of the fluctuating light cycle (Fig. 4B), indicating a lower PSII/PSI ratio in the absence of PSB33. Similarly, the quantum yield of PSII and PSI in wild-type and *psb33* mutant plants was recorded for 2 h in 30 min cycles of LL1 (70 PFD), HL (850 PFD), LL2 (70 PFD), and finally darkness. In correspondence to unaltered PsaB protein levels, the activity of PSI was unaffected between wild-type and mutant plants while the efficiency of PSII was markedly lower after the HL period in mutant plants (see Supplementary Fig. S2 at *JXB* online). Finally, the specific levels of Lhcb1, Lhcb2, and their phosphorylated forms (P-Lhcb1 and P-Lhcb2, respectively) (Fig. 4C) (Leoni *et al.*, 2013) were assessed. The Lhcb1 or Lhcb2 protein levels did not show significant differences between wild-type and *psb33* in any light condition. The phosphorylated forms of Lhcb1 and Lhcb2 were detectable after the Da1 period in *psb33*, although to a lesser extent than in wild-type plants. After the first low-light treatment (LL1), the levels of Lhcb1 and Lhcb2 phosphorylation were lower than after Da1 in *psb33*, the converse of the induction visible in wild-type plants. Following the high- and low-light treatments, the levels of Lhcb phosphorylation in *psb33* plants continued to decrease during each light interval, while reversible phosphorylation states were detected in wild-type plants (Fig. 4C). Interestingly after a 2 h dark period both for the wild-type and *psb33* plants, the Lhcb phosphorylation levels returned to the original Da1 level, suggesting that STN7 is functional also in *psb33* (Fig. 4C). As a lower PSII/PSI ratio could explain the defective phosphorylation, we artificially induced the excitation of PSII or PSI by illuminating plants with a light series consisting of 30-min periods of Dark, Red, Red+Far Red (Fr), Red, and finally Red+Fr (Supplementary Fig. S3). In contrast to the results observed in response to our series of fluctuating light intensities, reversible phosphorylations of Lhcb1 and Lhcb2 were functional in *psb33* mutant plants upon Red/Fr treatments, although to a lesser extent than in wild-type plants, indicating that a light-controlled mechanism of the kinase is impaired in the absence of the PSB33 protein during natural fluctuating lights.

### Relative amounts of photosystem II, cytochrome *b*_6_*f* and photosystem I in *psb33* and wild-type plants

**Fig. 5.**
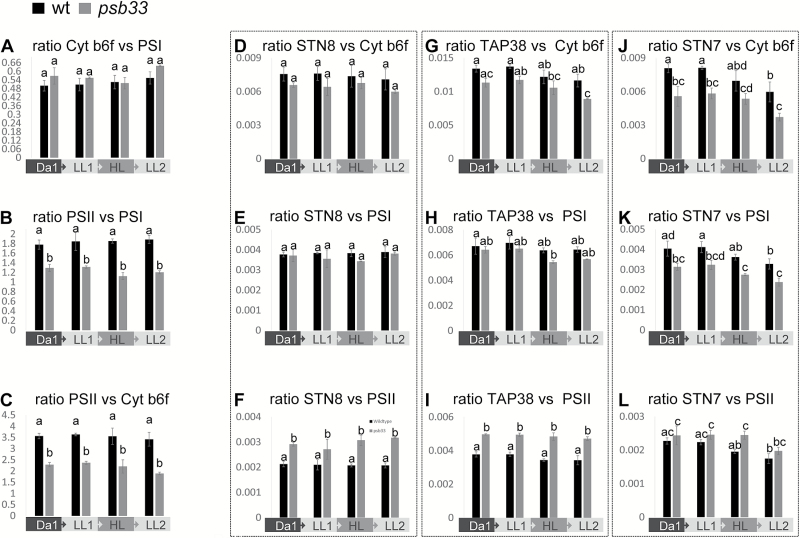
PSII and STN7 proteins are depleted in *psb33* plants. Relative quantification of the major thylakoid protein complexes PSII, PSI and Cyt *b*_6_*f* and of the same complexes against STN8, TAP38, and STN7 by means of SRM from thylakoids of wild-type and *psb33* plants sampled at the end of 16 consecutive hours of dark (Da1), 2 h of low light (LL1), 2 h of high light (HL), and another 2 h of low light (LL2). The sum of the intensities in terms of the spectral counts of at least three proteotypic peptides for each protein was used, while the numbers for PSII, PSI, and Cyt *b*_6_*f* are the results of the sum of three peptides from CP47 and from D2, from PsaA and from PsaB, and from Cyt *b*_6_ and from Cyt *f*, respectively. Bars indicate averages of two biological replicates (for *psb33*) and three biological replicates (wt), each with two technical replicates. Letters above bars indicate statistically significant differences determined by one-way ANOVA with Holm–Sidak’s *post hoc* test (*P*<0.05). (A) SRM relative quantification of the ratios between Cyt *b*_6_*f* and PSI, demonstrating no differences between genotypes and light treatments. (B) SRM relative quantification of the ratios between PSII and PSI and (C) PSII and Cyt *b*_6_*f*, demonstrating a significantly lower amount of PSII in *psb33* plants. (D) SRM relative quantification of the ratios of STN8 *versus* Cyt *b*_6_*f*, (E) *versus* PSI and (F) *versus* PSII, demonstrating that the STN8 level is not affected by a lower PSII amount. (G) SRM relative quantification of the ratios of TAP38 *versus* Cyt *b*_6_*f*, (H) *versus* PSI and (I) *versus* PSII, showing no major differences, as in the case of STN8. (J) SRM relative quantification of the ratios between STN7 and Cyt *b*_6_*f* (K) and PSI, demonstrating significant degradation of STN7 after HL treatment in both genotypes, and (L) the ratio between STN7 and PSII, demonstrating a lower amount of STN7 when PSII is depleted in *psb33* samples, unlike STN8 and TAP38.

As the lack of PSB33 affects PSII both with respect to phosphorylation dynamics (Fig. 4A) and at the protein level (Fig. 4B), we next analysed the consequences of the lack of PSB33 on other thylakoid complexes and regulatory proteins. To gain an accurate picture of the relative amounts of PSII, Cyt *b*_6_*f* and PSI and of the kinases STN7 and STN8, the phosphatase TAP38 and PSB33 itself, a relative quantification by mass spectrometry analysis was performed on wild-type and *psb33* thylakoid proteins isolated from plants treated with the fluctuating light cycle as described above, excluding the last dark period (Fig. 5 and Supplementary Fig. S4). The relative amounts of the major thylakoid protein complexes in the *psb33* mutant, in comparison with wild-type membranes, were assayed by selected reaction monitoring (SRM) as described in (Trotta *et al.*, 2016). At least three unique peptides from two subunits of PSII (D2 and CP47), PSI (PsaA and PsaB), and Cyt *b*_6_*f* (Cyt *f* and Cyt *b*_6_) and from STN7, STN8, TAP38, and PSB33 were used to calculate the ratios between the three thylakoid complexes and between the complexes and the above-mentioned regulatory proteins, based on spectral counts (intensity) (Trotta *et al.*, 2016). An advantage of using the SRM approach over standard immunoblotting is that all peptides are analysed in the same sample without the problem of overlapping signals. While the ratios between Cyt *b*_6_*f* and PSI were similar in all wild-type and *psb33* samples tested (Fig. 5A), the ratios PSII/PSI and PSII/Cyt *b*_6_*f* were clearly lower in *psb33* plants with respect to wild-type plants (Fig. 5B and C, respectively), in line with the results obtained with D1 immunoblotting (Fig. 4B). Intriguingly, the relative amounts of STN7, STN8, and TAP38 showed different behaviors: the STN8 and TAP38 levels were largely similar between wild-type and mutant samples when compared with Cyt *b*_6_*f* (Fig. 5D, G) and PSI (Fig. 5E, H), and they were, consequently, higher in *psb33* plants when compared with PSII (Fig. 5F, I). Conversely, the amount of STN7 was clearly tied to the amount of PSII (Fig. 5l); both the STN7 and PSII proteins were present in low amounts, relative to PSI and Cyt *b*_6_*f*, in the thylakoids of the *psb33* plants (Fig. 5J, K). Moreover, STN7 showed a degradation pattern with increasing light intensity, which did not revert with the second low-light period in wild-type plants, in line with previous results (Trotta *et al.*, 2016), or in the *psb33* plants (Fig. 5J, K, L). Instead, the ratio between PSB33 and the various thylakoid protein complexes did not show a clear change in wild-type plants in any of the light conditions applied (Supplementary Fig. S4A, B, C).

### Lhcb1 and Lhcb2 distribution is unaltered in BN-PAGE

**Fig. 6.**
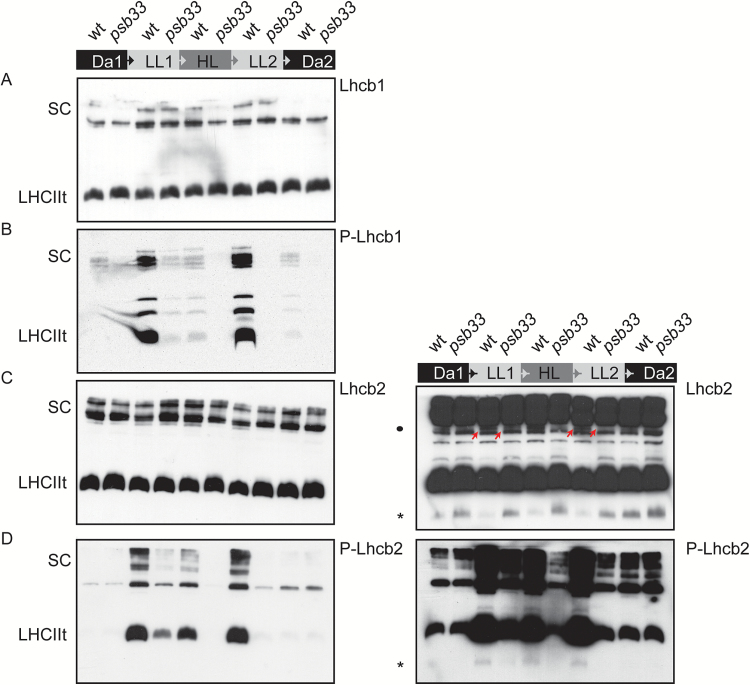
Native distribution of the major subunits of light harvesting complex II and their phosphorylated forms. Digitonin-solubilized thylakoid pigment-protein extracts isolated from wild-type or *psb33* plants exposed to the same dark–light cycle described in Fig. 1 were separated by lpBN gel and immunodetected against (A) Lhcb1, (B) P-Lhcb1, and (C) Lhcb2. The right panel shows an overexposed blot of Lhcb2, and the asterisk indicates a low molecular mass band that corresponds to its opposite in (D) (asterisk). (D) P-Lhcb2. The right panel shows an overexposed blot of P-Lhcb2, and the asterisk indicates a low molecular mass band. (This figure is available in color at *JXB* online.)

While the amounts of LHCII from whole thylakoids seemed to be equal in the single-dimension gel (Fig. 4), the distribution of the LHCII antenna proteins in monomers, trimers and protein complexes could be influenced by phosphorylation. Thus, the distributions of Lhcb1 and Lhcb2 in the non-appressed thylakoid regions of *psb33* were analysed from the same dark–light–dark cycle using antibodies against Lchb1, P-Lhcb1, Lhcb2, and P-Lhcb2 (Fig. 6). The distribution of Lhcb1 did not show any drastic differences between wild-type and *psb33* plants (Fig. 6A). In contrast, Lhcb1 phosphorylation was strongly induced only in wild-type plants under low light (Fig. 6B), as also shown by one-dimensional SDS-PAGE (Fig. 4C). The level of P-Lhcb1 was drastically reduced in *psb33* plants compared with the wild-type in LL1, and no detectable phosphorylation in HL, LL2, or Da2 was found in *psb33* plants (Fig. 6B). The distribution of Lhcb2 protein in *psb33* was similar to that of the wild-type, with Lhcb2 being mostly present as a trimer and as a part of the supercomplexes (Fig. 6C). Again, the exposure of plants to low light induced the prominent phosphorylation of Lhcb2 in the wild-type (Fig. 6D). Phosphorylated Lhcb2 was localized to LHCII trimers, to a state-transition-specific complex and to the supercomplexes. In contrast, in *psb33* plants, Lhcb2 phosphorylation was only marginal, but the residual amount of phosphorylated Lhcb2 was present in the same complexes as in the wild-type (Fig. 6D). Intriguingly, longer exposures of the Lhcb2 immunoblots revealed an additional band with low molecular mass in wild-type samples that was visible in darkness but depleted upon exposure to any light intensity (Fig. 6C right panel asterisk), along with an additional immunosignal in a higher molecular mass region (Fig. 6C right panel bullet and indicated with arrows). Conversely, the same low molecular mass band was detected by the P-Lhcb2 antibody in light-treated samples in the wild-type (Fig. 6D right panel asterisk). In *psb33*, Lhcb2 remained in this low molecular mass complex despite the exposure to low light, which correlated with low phosphorylation of the same band.

The data above indicated that the *psb33* mutant is unable to properly phosphorylate Lhcbs and PSII core proteins under changing light intensities, despite having seemingly functional STN7. To investigate if a defective phosphorylation affects the distribution of Lhcbs in *psb33* and if PSB33 determines substrate specificity of STN7-mediated phosphorylation, we evaluated the dynamics of the Lhcb1/Lhcb2 pair with respect to the three thylakoid protein complexes PSII, Cyt *b*_6_*f* and PSI, using the same quantification by mass spectrometry analysis approach (see Supplementary Fig. S4D–F). No striking differences could be observed in the different light treatments. However, it was also possible to measure the relative percentage of phosphothreonine for Lhcb1, which appeared to be markedly lower in *psb33*, following the same pattern observed with western blots in Fig. 4. In contrast, no difference between wild-type and *psb33* plants was detected in the serine phosphorylation of either Lhcb1 or Lhcb2, providing evidence that two distinct mechanisms exist for the phosphorylation of Lhcb threonine and serine residues (Supplementary Fig. S4G–I).

### PSII depletion in *psb33* plants is affected by the spectral quality of light and the design of the fluctuating light treatment

**Fig. 7.**
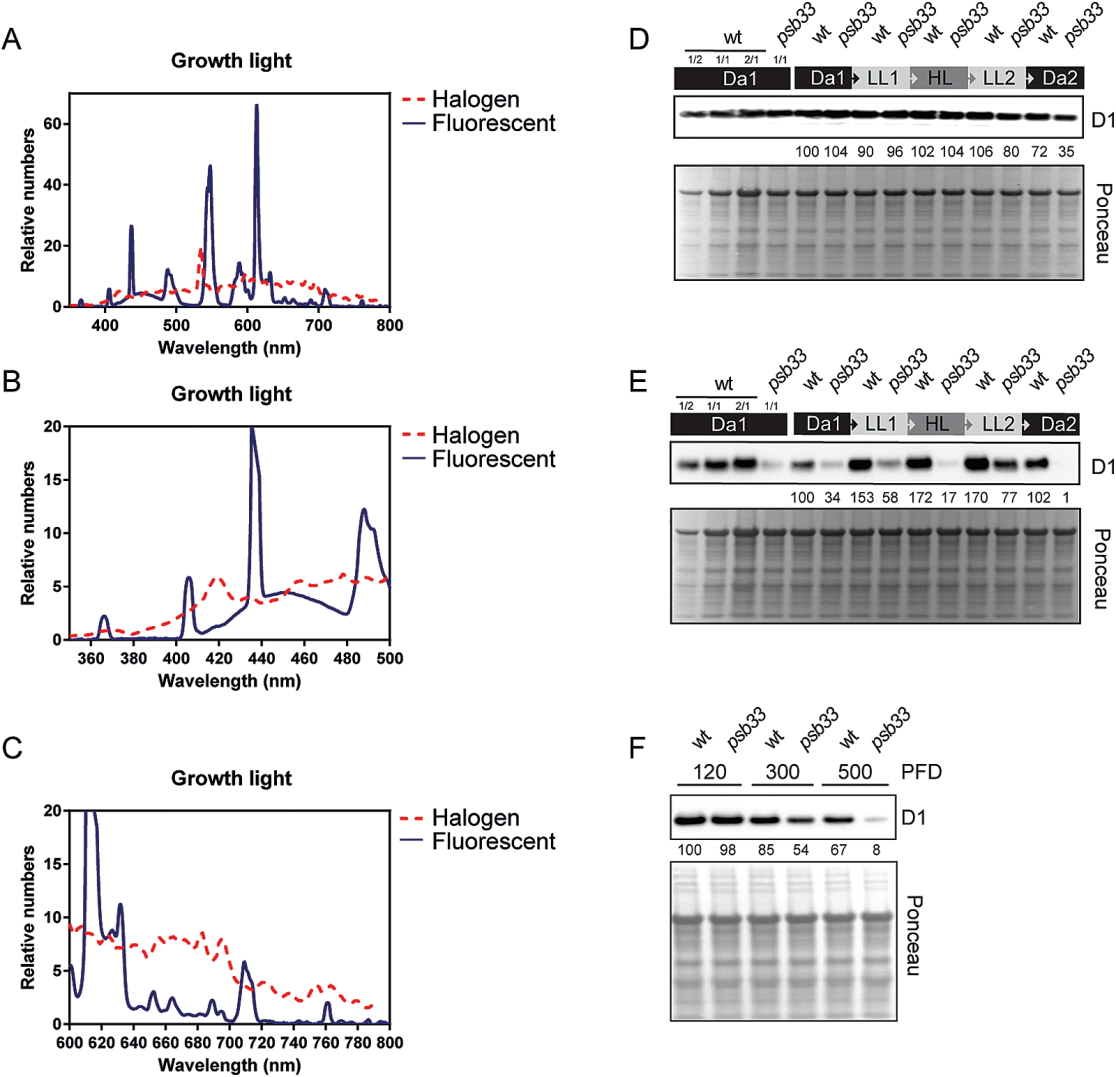
Effects of light spectrum and design of the fluctuating light treatment on PSII depletion. Comparison of the wavelength spectra of metal halide and fluorescent lamps and the effects of fluorescent lamps on D1 protein level before and after two consecutive treatments with fluctuating light (16 h of dark (Da1), followed by 2 h of low light (LL1), 2 h of high light (HL), another 2 h of low light (LL2), and finally 8 h of dark (Da2). (A) Normalized comparison of light spectra from growth chambers equipped with metal halide lamp (dashed line) and fluorescent lamp (solid line) light sources and (B) magnification of the differences between the spectra in the blue and (C) red regions. (D) D1 immunoblot with whole-leaf protein extracts from wild-type (wt) and *psb33* plants, demonstrating that PSII is not constitutively depleted in *psb33* plants grown under fluorescent lamps but is still more susceptible to degradation at the end of the first fluctuating light period and (E) is even more severely affected by a second fluctuating light period. Quantitative levels averaged from three individual repetitions of D1 protein levels, which have been normalized to the Da1 D1 level, are represented as numeric values below the D1 immunoblot in (D) and (E). A Ponceau-stained PVDF membrane is shown to demonstrate equal loading. (F) Effect of incubation of leaves with intact petioles from wt and *psb33* plants, incubated overnight in the dark with 1 mM lincomycin and then transferred to the 120, 300, or 500 PFD for 2 h, on the degradation of D1 protein, as detected by immunoblot with D1 antibody. A Ponceau-stained PVDF membrane is shown to demonstrate equal loading. (This figure is available in color at *JXB* online.)

The striking influence of the PSB33 protein on PSII abundance prompted us to investigate whether the light quality, affecting the relative distribution of light energy to PSII and PSI (light enriched in blue and red, favoring PSII excitation), would modify PSII dynamics in the absence of PSB33. To this end, wild-type and *psb33* plants were grown under fluorescent lamps, providing a different light spectrum from the metal halide lamps used in our previous experiments (Fig. 7A, B, C; Supplementary Fig. S5). Plants were treated with the same fluctuating light cycle described above, and thylakoids were isolated and analysed. As visible in the immunoblot (Fig. 7D), the levels of PSII D1 protein were similar in *psb33* and wild-type plants from the beginning of the light cycle until the end of the HL illumination. During the LL2 and Da2 periods, a substantial decrease in the amount of the D1 protein occurred in the *psb33* mutant. To gain a more comprehensive picture of the dynamics of PSII under fluorescent lamps, the wild-type and *psb33* plants were allowed to dark-acclimate for 8 h after the first fluctuating light cycle and were subsequently treated with a complete second cycle of the fluctuating lights (Fig. 7E). Notably, after an extensive dark period of 8 h, the wild-type plants adjusted their PSII D1 protein upon light exposure, in contrast to the first days of fluctuating light exposure, while the *psb33* plants showed a severe decrease in PSII D1 protein abundance after the dark period (Fig. 7E). Furthermore, after the HL period, the D1 protein was undetectable, and after appearing again in LL2, it was again undetectable after Da2 in the mutant. This result indicated that the *psb33* mutant was capable of the repair and/or biogenesis of new PSII complexes during the light period.

To study the relationship between the extent of damage to D1 and a possible role of PSB33 in the repair cycle, the leaves from wild-type and *psb33* plants were incubated with lincomycin to inhibit the translation of the D1 protein and were subsequently exposed to growth, moderate, and high light under fluorescent lamps (Fig. 7F). Moderate and high light induced severe damage to the D1 of the *psb33* plants and demonstrated that the effect on PSII was linked to the extreme light sensitivity of the *psb33* plants rather than to impairment of the PSII repair cycle in light (Fig. 7F). Finally, to rule out the possibility that the dynamics of the D1 protein in *psb33* plants were linked to the lack of LHCII phosphorylation, the *stn7* plants were treated with the same fluctuating cycle, and the level of D1 protein was analysed by western blotting. No degradation of D1 could be observed in the *stn7* plants, indicating that the accelerated D1 degradation typical for *psb33* is not due to the lack of state transition (see Supplementary Fig. S6 at *JXB* online).

## Discussion

The acclimation of the photosynthetic machinery to changing light conditions requires the dynamic mobility, disassembly and reassembly of thylakoid protein complexes and various cofactors. This requirement is especially true for PSII, and many subunits are involved in the regulation of this complex (Järvi *et al.*, 2015; Mulo *et al.*, 2008; Nickelsen and Rengstl, 2013). One such subunit, the PSB33 protein, was previously shown to be involved in the accomplishment of state transitions, and the loss of PSB33 resulted in a high light-sensitive phenotype (Fristedt *et al.*, 2015). Here, we show that, similar to the *stn7* mutant (Tikkanen *et al.*, 2010), prolonged fluctuating light induces a severe stunted growth phenotype in *psb33* plants. However, while *stn7* showed a 50% decrease in biomass, the biomass of the *psb33* mutant was reduced by up to 85% compared with wild-type plants under similar conditions (Fig. 1A). This result clearly indicates that the *psb33* phenotype is unlikely to be explained by problems in state transitions alone. To deepen the understanding of PSB33’s role in the light acclimation process of PSII in Arabidopsis, we examined *psb33* at different irradiation levels during a fluctuating light cycle using a combination of biochemical and proteomics tools.

### PSB33 is primarily located in non-appressed thylakoid regions

In higher plants, the heterogeneity of the thylakoid membrane is fundamentally important for acclimation and maintenance of the photosynthetic protein complexes. In recent years, the detailed functions of many thylakoid proteins have been determined, and it is clear that functional/locational specificity exists. For example, the FtsH and Deg proteases are located in the non-appressed membranes (Lindahl *et al.*, 1996; Haußühl *et al.*, 2001), and the proteolytic accessibility of damaged D1 is controlled in these parts of the membrane (Aro *et al.*, 2005). Strikingly, here, we demonstrate by thylakoid fractionation that PSB33 resides peripherally in the non-appressed thylakoid domain, particularly in stroma lamellae (Fig. 2). This finding opens up questions about PSB33’s functional role, which clearly expands beyond maintaining PSII stability in the grana. Consequently, we performed a detailed membrane location analysis of appressed thylakoid regions and show that most of PSB33 aligns with the LHCII monomer or in the free-protein fraction (Fig. 3A). More importantly, in non-appressed regions, PSB33 comigrated with PSII monomers and with an as yet uncharacterized complex that has a molecular mass between PSII monomers and Cyt *b*_6_*f* (Fig. 3B). As a recent report proposed interaction between the EX1 protein and PSB33 within grana margins (Wang *et al.*, 2016*b*), an appealing speculation would be that this undisclosed complex holds the interaction between PSB33 and EX1. The EX1 proteins are suggested to sense the production of reactive oxygen species (Wang *et al.*, 2016*b*). Interestingly, in the coimmunoprecipitation (Co-IP) with EX1 and PSB33, several LHCII, LHCI, and FtsH proteins were found (Wang *et al.*, 2016*b*). The PSB33 association with this complex may be light regulated, as the intensity of the spot is stronger under dark and is diminished under higher light, whereas PSB33 seems to relocate to the free protein region in the BN gels.

### Regulation of thylakoid protein phosphorylation is defective in *psb33*

Consistent with missing state transitions, the typical and reversible PSII–LHCII phosphorylation dynamics upon short-term changes in light intensity were completely lost in *psb33* plants (Figs 4 and 5). Analysis of the thylakoid protein phosphorylation revealed an irreversible effect after high-light treatment in *psb33* plants that are unable to control LHCII and PSII phosphorylation (Fig. 4). A possibility would be that the plastoquinone (PQ) pool redox state is affected in *psb33*, with obvious downstream consequences. A misregulated electron transport chain could explain why the STN7 target is not phosphorylated after the fluctuating light treatment. In this respect, it is important to note that the phosphorylation of STN7 substrates is similar to the wild-type after a long dark acclimation of *psb33* plants, while a lack of kinase activity would have resulted in the complete absence of thylakoid protein phosphorylation. More importantly, artificial red light induced LHCII phosphorylation in the plants lacking PSB33, clearly indicating that STN7 is functional in the *psb33* plants (see Supplementary Fig. S3). Furthermore, the specific upstream effect of the lack of PSB33 with respect to STN7 and STN8 activities could be inferred by the fact that *psb33* and wild-type plants display similar levels of a LHCII serine phosphorylation, which has not been shown to be STN7/STN8 dependent (Roitinger *et al.*, 2015) (Supplementary Fig. S4H). Nevertheless, we cannot exclude that the decreased phosphorylation of PSII core proteins is further influenced by the decrease in the amount of the D1 protein.

Upon more detailed characterization of the stoichiometry of thylakoid protein complexes, we observed an overall depletion of PSII in *psb33* thylakoids when plants were grown in a climate chamber equipped with metal halide lamps that favor PSII excitation (Fig. 4 and Supplementary Fig. S5). Furthermore, the relative quantification of the kinases STN7 and STN8 and the TAP38 phosphatase, essential for proper state transitions, indicated that PSII depletion was accompanied by a similar depletion of STN7 but not of STN8 or TAP38 (Fig. 5). Most interestingly, this result suggests a previously undescribed coregulation of PSII core proteins and STN7.

### Why is PSII D1 protein so exceptionally susceptible to degradation in PSB33-lacking plants?

The light sensitivity of *psb33* plants was previously explained by unstable PSII supercomplexes (Fristedt *et al.*, 2015). Here, we demonstrate that the PSII core protein D1 is exceptionally sensitive to degradation in the *psb33* mutant. Using quantitative mass spectrometry, we observed that PSII was already markedly depleted in the *psb33* plants at the first dark measuring point compared with wild-type plants when grown under light conditions favoring PSII excitation (Fig. 5). After disclosing the reason for differential depletion of PSII depending on the light source during growth, we could segregate the effect of fluctuating light from the effect of light quality on PSII. When the light source for growth of the *psb33* plants was shifted to fluorescent tubes to alleviate the excitation pressure on PSII (Fig. 7), the consecutive fluctuating light series were still severely affecting the PSII level in *psb33*. Strikingly, the largest decrease of D1 in the *psb33* mutant was observed during the following dark period (Fig. 7). In wild-type plants, such a loss of D1 is compensated for by repair and/or new PSII biogenesis.

The strong dependence of *psb33* D1 protein stability on the light spectra, upon plant growth, as well as the dark intervals between the light exposures are particularly interesting considering the recent publication by Cruz *et al.* (2016) demonstrating an extremely patchy phenotype for *psb33*. *psb33* plants were described as having an ‘emergent’ phenotype observed under the dynamic growth environment used. In short, during a 5 days period of dynamic growth light, *psb33* plants showed identical photosynthetic parameters to wild-type plants on days 1 and 2, but then showed a severely decreased maximal PSII quantum efficiency on days 3 and 4. The loss of PSII quantum efficiency in *psb33* recovered on day 5, but subsequent illumination with fluctuating light on day 5 once again induced reduced PSII quantum efficiency in the *psb33* plants (Cruz *et al.*, 2016). Our results and the results from Cruz *et al*. clearly indicate that PSB33 plays a key role in PSII photosynthetic capacity under fluctuating light conditions but appears less important under stable light conditions.

As described before, mutants showing a strong conditional phenotype similar to *psb33* include the PSII assembly factors TLP18.3 (Sirpiö *et al.*, 2007) and MET1 (Bhuiyan *et al.*, 2015), along with the state transition kinase STN7 (Bellafiore *et al.*, 2005) and the thylakoid PGR5 protein (Suorsa *et al.*, 2013). TLP18.3 is located in the lumen, while MET1 is located in the non-appressed membranes. Both of these proteins are required for the PSII repair cycle under fluctuating growth light, and the PSII supercomplex formation is reduced but not abolished in the knockout mutants. It is possible that both TLP18.3 and MET1 are involved in making the PSII assembly/disassembly process more efficient but are not strictly required. Even more severe conditional phenotypes are observed for STN7 and PGR5 (lethal) under fluctuating light conditions. However, these mutants are different and have specific functions in the protection of PSI under fluctuating light conditions (Tikkanen *et al.*, 2010; Suorsa *et al.*, 2013; Tiwari *et al.*, 2016). Thus, the fluctuating-light mutants characterized so far fall into two categories: those specifically affecting PSII and those having a damaging effect on PSI.

It is clear from our results that PSB33 is not involved in the protection of PSI whilst its role in the sustenance of PSII is unambiguous. Nevertheless, it is unlikely that PSB33 would directly assist in the assembly or repair of PSII. First, PSB33 does not harbor any typical domains for ‘assembly’ factors, such as chaperones, foldases, PPIases or proteases. PSB33, however, contains a chlorophyll *a*/*b*-binding motif, just like the PsbS protein, but the function of such a domain still remains elusive. Recently, it was shown that a single chlorophyll *a* molecule in Cyt *b*_6_*f* may act as a redox sensor for the redox state of the PQ pool to facilitate the state transition (Vladkova, 2016). It is possible that PSB33 has a similar sensory role; thus, a detailed study of the PSB33 chlorophyll binding motif may give a more detailed view of its regulatory function. Second, there are PSII supercomplexes present in *psb33*, but they are very unstable, especially under high light conditions (see Supplementary Fig. S7). Indeed, the thylakoid phosphorylation and the amount of the PSII D1 protein oscillate according to light intensity and quality in plants lacking the PSB33 protein (Figs 4A, B and 7D, E). This relationship provides compelling evidence that the proper activation/deactivation of both the STN7 kinase and the D1 protease are out of phase in the absence of PSB33. It is conceivable that PSB33 has a unique role in adjusting such a regulation according to the redox status of PSII, possibly in association with an EX1-containing complex.

In conclusion, we show that PSB33 is required for proper PSII quality control, especially under fluctuating light conditions. Since the discovery of the STN7 and STN8 kinases and corresponding phosphatases, responsible for the phosphorylation status of LHCII and PSII core proteins, respectively, these protein modifications have been thought to be the main trigger for LHCII rearrangements and the quality control of PSII to balance and maintain photosynthesis. Here, we demonstrate that PSB33 has an even more imperative role in PSII quality control. Consideration of the present results together with the patchy phenotype of *psb33* that was linked by Cruz *et al* (2016) to a nocturnal event raises a possibility of two distinct mechanisms, a diurnal one and a nocturnal one, that need to be tightly adjusted under various light conditions to optimize and maintain photosynthesis. We show that PSB33 is likely to play a role in both of these processes. Undeniably, PSB33 is fundamentally important for the proper function of photosynthesis in higher plants, and the details surrounding this protein will be investigated in great detail in the future.

## Supplementary data

Supplementary data are available at *JXB* online.

Fig. S1. PSB33 is completely solubilized after the digitonin treatment of the thylakoids.

Fig. S2. Photosynthetic quantum efficiency dynamics from wild-type and *psb33* mutant plants.

Fig. S3. Dynamics of thylakoid membrane phosphoproteins in response to artificially induced state transition conditions in wild-type and *psb33*.

Fig. S4. Relative quantification of the ratio between PSB33 or the sum of lhcb1+lhcb2 and the major thylakoid protein complexes PSII, PSI and Cyt *b*_6_*f* and the percentages of phosphorylation of two different phosphosites in lhcb1 and 2 by SRM.

Fig. S5. Comparison of the wavelength spectra of metal halide and fluorescent lamps with respect to PSII and PSI activity and absorption of chlorophyll *a* and *b*.

Fig. S6. *stn7* D1 protein is stable under fluctuating light.

Fig. S7. PSII supercomplexes in *psb33* are unstable under light conditions.

Table S1. Peptides and related transitions detected in wild-type plants and the *psb33* mutant belonging to the proteins indicated in Fig. 5 and Supplementary Fig. S4.

## Author contributions

RF, AT, MS, AN, and BL designed and performed experiments. All authors contributed to the analysis of results and writing of the manuscript.

## Supplementary Material

Supplementary_Figures_S1_S7_Table_S1
