# Supplementary material for: PSB33 sustains photosystem II D1 protein under fluctuating light conditions
Source: J Exp Bot. 2017 Jul 26;68(15):4281–93. doi: 10.1093/jxb/erx218 (PMC5853261; doi:10.1093/jxb/erx218)
Supplement: Supplementary_Figures_S1_S7_Table_S1 [file erx218_suppl_supplementary_figures_s1_s7_table_s1.pdf]

Supplemental figure 1

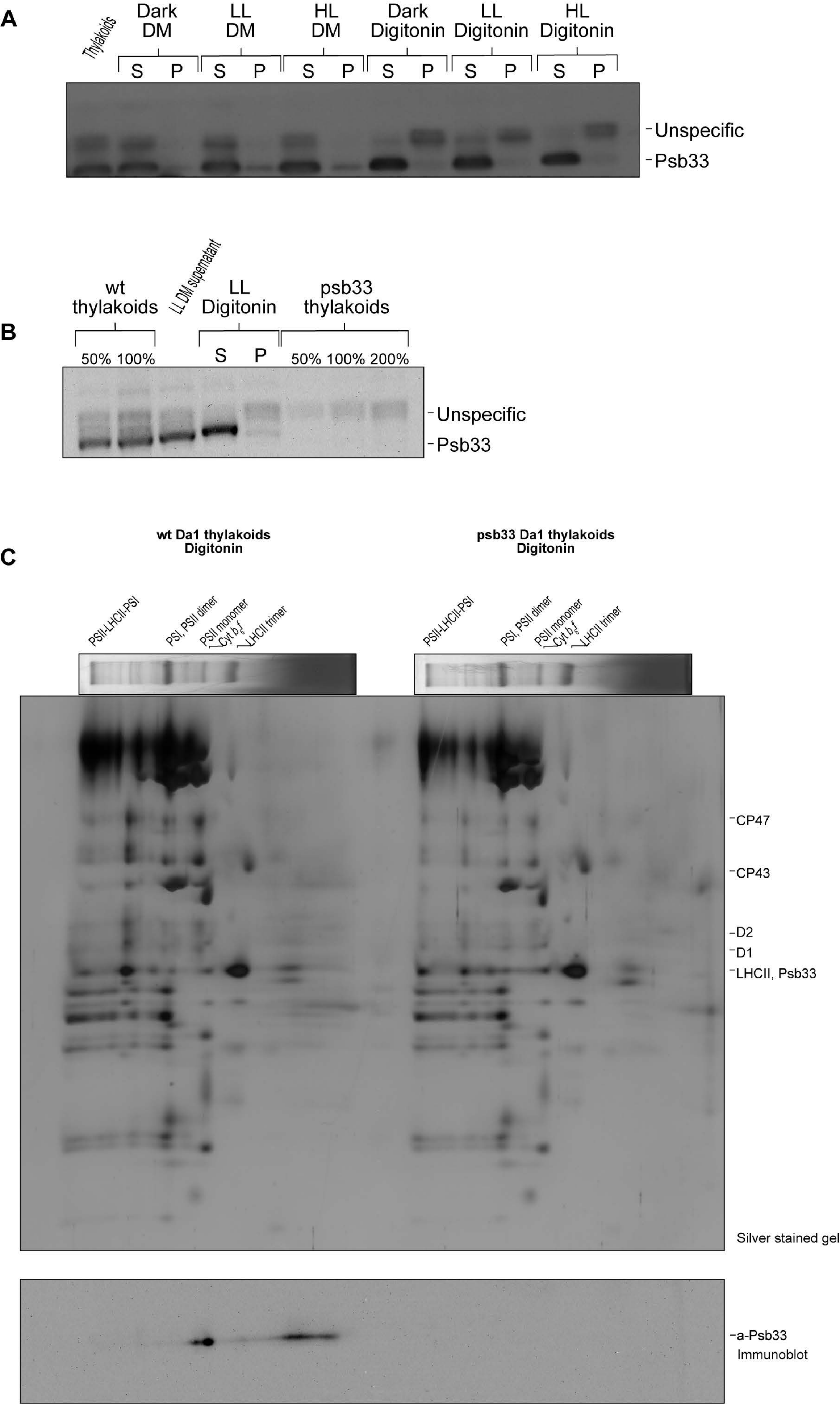

Supplemental figure 2

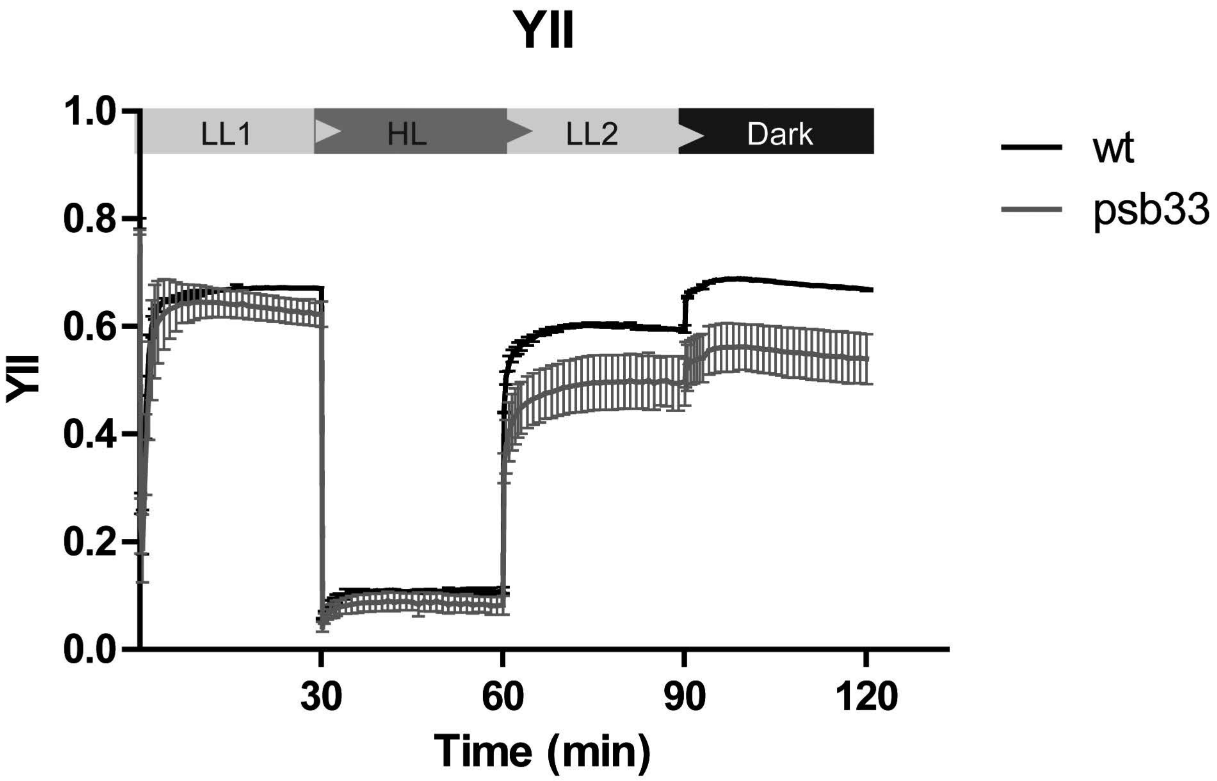

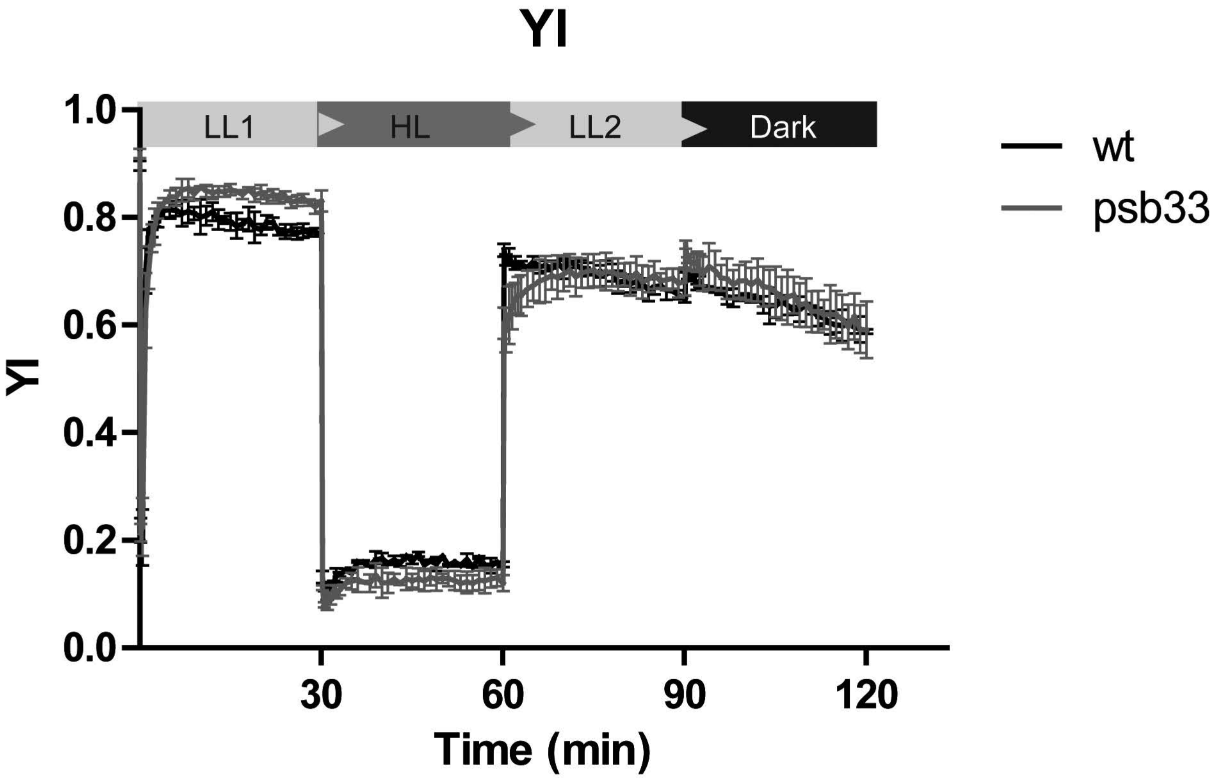

Supplemental figure 3

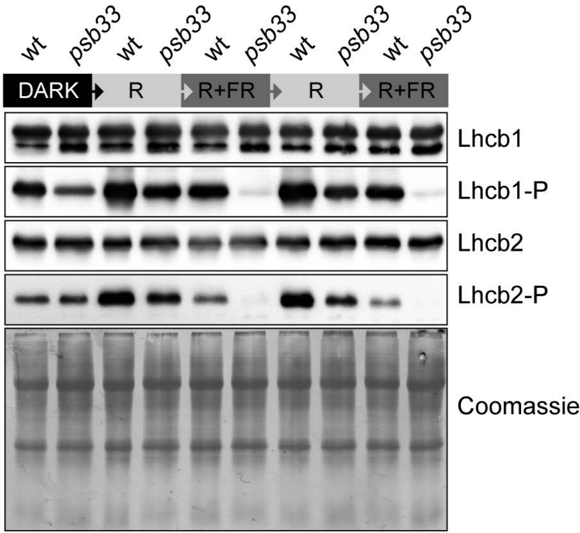

Supplemental figure 4

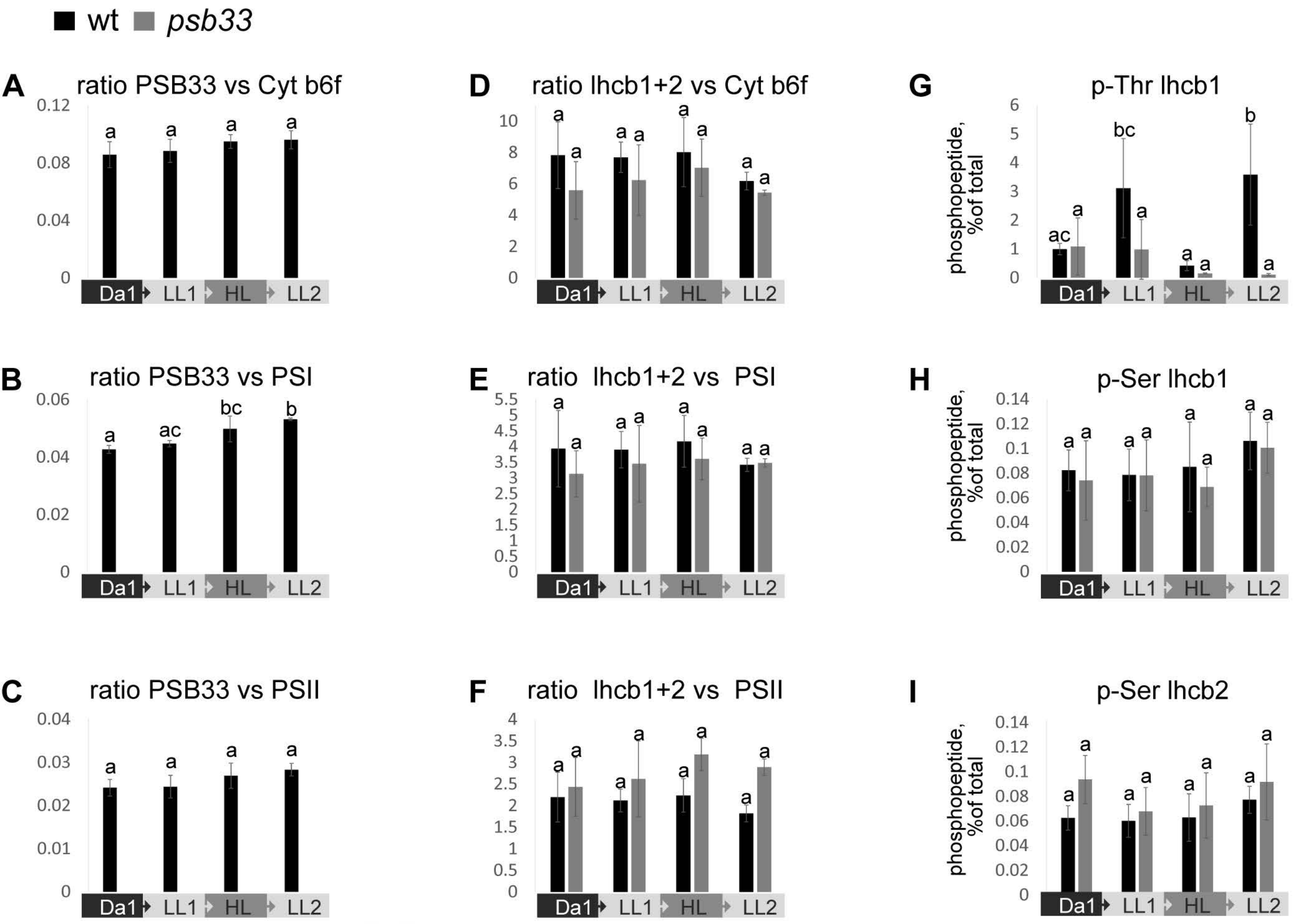

Supplemental figure 5

A

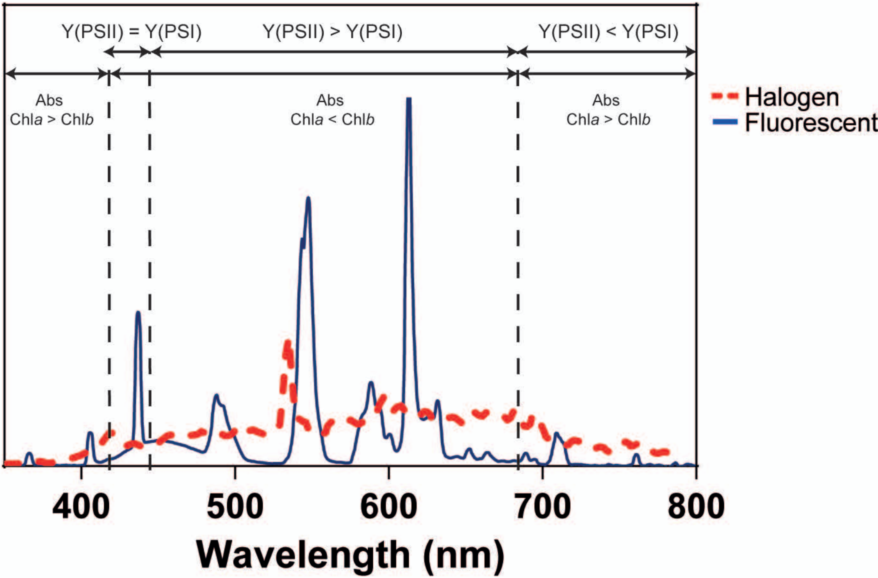

B

Total distribution of light (percentage) in different regions of wavelength favoring either absorption of chlorophyll *a* (300-420nm; 680-800nm) or chlorophyll *b* (420-680nm)

	300-420nm	420-680nm	680-800nm
Fluoresencent	2%	93%	5%
Halogen	5%	76%	19%

Supplemental figure 6

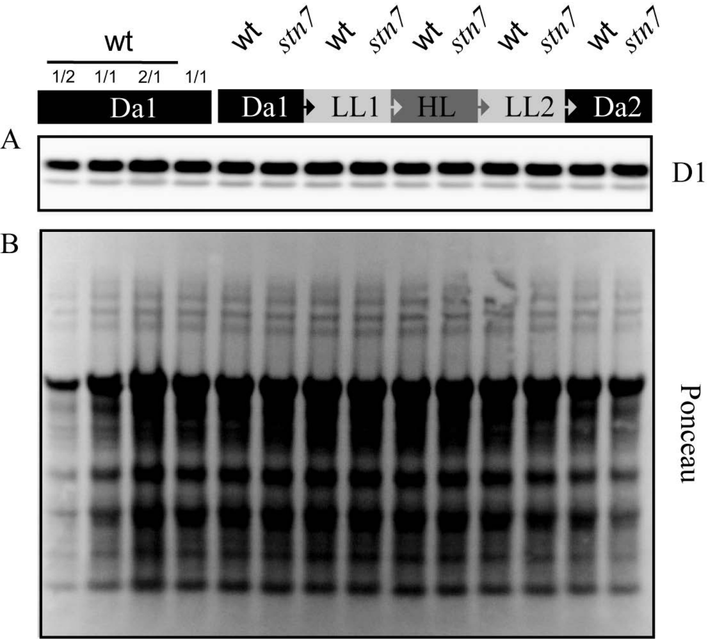

Supplemental figure 7

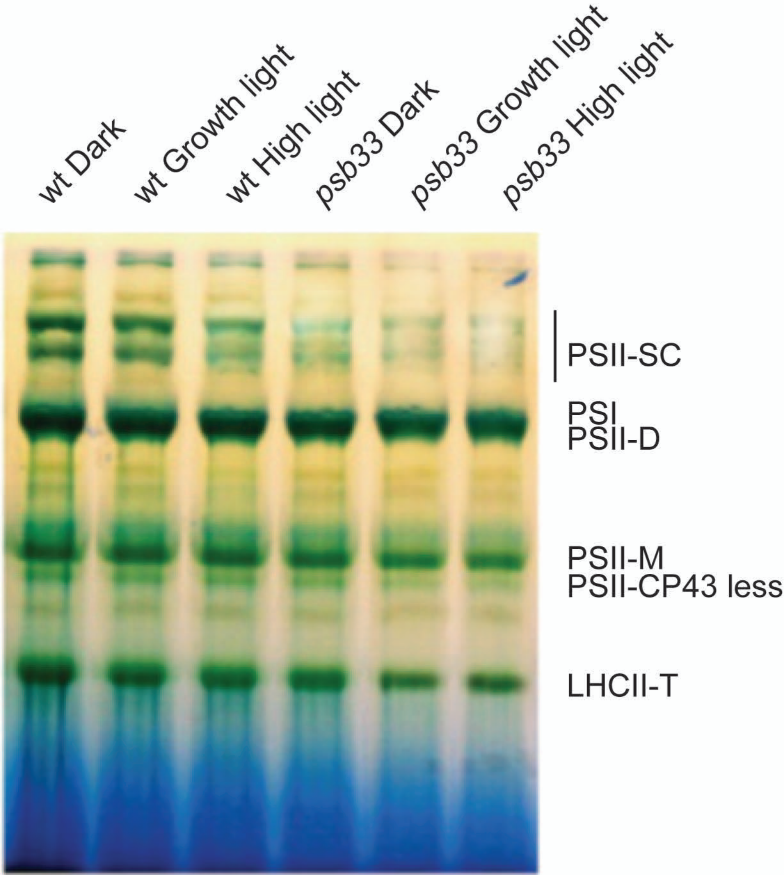

Supplemental table 1: List of peptides and related transitions used to detect the peptides belonging to the proteins indicated in Figure 5 and Supplemental Figure 3. Highlighted in yellow are the transitions used to relatively quantify ratios between thylakoid proteins and to raltively quantify the phosphorylation levels of Lhcb1

AGI code	Uniprot Protein Acces	Protein Description	Peptide Modified Sequence	Precursor Mz	Precursor Charge	Product Mz	Product Charge	Fragment Ion	Cleavage A	Protein Description	Protein Acc
ATCG00270.1	P56761	PSBD | photosystem II reaction center protein D	AYDFVSQEIR	614,303852	2	993,499986	1	y8	D	PSBD | photosystem II reaction center protein D	P56761
						632,336215	1	y5	S	PSBD | photosystem II reaction center protein D	P56761
						350,134661	1	b3	D	PSBD | photosystem II reaction center protein D	P56761
ATCG00270.1	P56761	PSBD | photosystem II reaction center protein D	AAEDPEFETFYTK	774,348654	2	1161,546267	1	y9	P	PSBD | photosystem II reaction center protein D	P56761
						935,45091	1	y7	F	PSBD | photosystem II reaction center protein D	P56761
						659,339903	1	y5	T	PSBD | photosystem II reaction center protein D	P56761
ATCG00270.1	P56761	PSBD | photosystem II reaction center protein D	AAEDPEFETFYTKNILLNEGIR	857,430621	3	814,478128	1	y7	L	PSBD | photosystem II reaction center protein D	P56761
						701,394064	1	y6	L	PSBD | photosystem II reaction center protein D	P56761
						588,31	1	y5	N	PSBD | photosystem II reaction center protein D	P56761
ATCG00270.1	P56761	PSBD | photosystem II reaction center protein D	NILLNEGIR	521,306198	2	814,478128	1	y7	L	PSBD | photosystem II reaction center protein D	P56761
						701,394064	1	y6	L	PSBD | photosystem II reaction center protein D	P56761
						588,31	1	y5	N	PSBD | photosystem II reaction center protein D	P56761
ATCG00680.1	P56777	PSBB | photosystem II reaction center protein B	RVSAGLAENQSLSEAWAK	958,994871	2	691,340966	1	y6	S	PSBB | photosystem II reaction center protein B	P56777
						475,266344	1	y4	A	PSBB | photosystem II reaction center protein B	P56777
						404,229231	1	y3	W	PSBB | photosystem II reaction center protein B	P56777
ATCG00680.1	P56777	PSBB | photosystem II reaction center protein B	RVSAGLAENQSLSEAWAK	639,665672	3	691,340966	1	y6	S	PSBB | photosystem II reaction center protein B	P56777
						584,351471	1	b6	L	PSBB | photosystem II reaction center protein B	P56777
						655,388585	1	b7	A	PSBB | photosystem II reaction center protein B	P56777
ATCG00680.1	P56777	PSBB | photosystem II reaction center protein B	VSAGLAENQSLSEAWAK	880,944315	2	1333,63827	1	y12	A	PSBB | photosystem II reaction center protein B	P56777
						1133,558563	1	y10	N	PSBB | photosystem II reaction center protein B	P56777
						891,457058	1	y8	S	PSBB | photosystem II reaction center protein B	P56777
						691,340966	1	y6	S	PSBB | photosystem II reaction center protein B	P56777
ATCG00680.1	P56777	PSBB | photosystem II reaction center protein B	VSAGLAENQSLSEAWAK	587,631969	3	891,457058	1	y8	S	PSBB | photosystem II reaction center protein B	P56777
						804,42503	1	y7	L	PSBB | photosystem II reaction center protein B	P56777
						691,340966	1	y6	S	PSBB | photosystem II reaction center protein B	P56777
						218,149918	1	y2	A	PSBB | photosystem II reaction center protein B	P56777
ATCG00680.1	P56777	PSBB | photosystem II reaction center protein B	IPEKLAFYDYIGNNPAK	651,675397	3	713,394064	1	y7	I	PSBB | photosystem II reaction center protein B	P56777
						600,31	1	y6	G	PSBB | photosystem II reaction center protein B	P56777
						315,202681	1	y3	P	PSBB | photosystem II reaction center protein B	P56777
ATCG00680.1	P56777	PSBB | photosystem II reaction center protein B	LAFYDYIGNNPAK	743,372266	2	991,484336	1	y9	D	PSBB | photosystem II reaction center protein B	P56777
						713,394064	1	y7	I	PSBB | photosystem II reaction center protein B	P56777
						600,31	1	y6	G	PSBB | photosystem II reaction center protein B	P56777
ATCG00680.1	P56777	PSBB | photosystem II reaction center protein B	LAFYDYIGNNPAK	495,917269	3	713,394064	1	y7	I	PSBB | photosystem II reaction center protein B	P56777
						600,31	1	y6	G	PSBB | photosystem II reaction center protein B	P56777
						315,202681	1	y3	P	PSBB | photosystem II reaction center protein B	P56777
ATCG00680.1	P56777	PSBB | photosystem II reaction center protein B	LAFYDYIGNNPAKGGLFR	672,682775	3	845,499198	1	y8	P	PSBB | photosystem II reaction center protein B	P56777
						549,314357	1	y5	G	PSBB | photosystem II reaction center protein B	P56777
						761,394064	2	y14	D	PSBB | photosystem II reaction center protein B	P56777
						565,806896	2	y11	G	PSBB | photosystem II reaction center protein B	P56777
ATCG00680.1	P56777	PSBB | photosystem II reaction center protein B	RAQLGEIFELDR	723,888615	2	1290,668842	1	y11	A	PSBB | photosystem II reaction center protein B	P56777
						1044,54727	1	b9	E	PSBB | photosystem II reaction center protein B	P56777
						1272,658277	1	b11	D	PSBB | photosystem II reaction center protein B	P56777
ATCG00680.1	P56777	PSBB | photosystem II reaction center protein B	RAQLGEIFELDR	482,928168	3	679,340966	1	y5	F	PSBB | photosystem II reaction center protein B	P56777
						532,272552	1	y4	E	PSBB | photosystem II reaction center protein B	P56777
						403,229959	1	y3	L	PSBB | photosystem II reaction center protein B	P56777
						655,352199	1	b6	E	PSBB | photosystem II reaction center protein B	P56777
ATCG00680.1	P56777	PSBB | photosystem II reaction center protein B	RAQLGEIFELDRATLK	620,682775	3	816,493778	1	y7	L	PSBB | photosystem II reaction center protein B	P56777
						655,352199	1	b6	E	PSBB | photosystem II reaction center protein B	P56777
						768,436263	1	b7	I	PSBB | photosystem II reaction center protein B	P56777
ATCG00680.1	P56777	PSBB | photosystem II reaction center protein B	AQLGEIFELDR	645,838059	2	1091,573151	1	y9	L	PSBB | photosystem II reaction center protein B	P56777
						978,489087	1	y8	G	PSBB | photosystem II reaction center protein B	P56777
						200,102967	1	b2	Q	PSBB | photosystem II reaction center protein B	P56777
ATCG00350.1	P56766	PSAA | Photosystem I, PsaA/PsaB protein	EIPLPHEFILNR	739,411726	2	1025,55269	1	y8	P	PSAA | Photosystem I, PsaA/PsaB protein	P56766
						791,441014	1	y6	E	PSAA | Photosystem I, PsaA/PsaB protein	P56766
						662,398421	1	y5	F	PSAA | Photosystem I, PsaA/PsaB protein	P56766
						513,279983	2	y8	P	PSAA | Photosystem I, PsaA/PsaB protein	P56766
ATCG00350.1	P56766	PSAA | Photosystem I, PsaA/PsaB protein	EIPLPHEFILNR	493,276909	3	791,441014	1	y6	E	PSAA | Photosystem I, PsaA/PsaB protein	P56766
						662,398421	1	y5	F	PSAA | Photosystem I, PsaA/PsaB protein	P56766
						513,279983	2	y8	P	PSAA | Photosystem I, PsaA/PsaB protein	P56766
						464,753601	2	y7	H	PSAA | Photosystem I, PsaA/PsaB protein	P56766
ATCG00350.1	P56766	PSAA | Photosystem I, PsaA/PsaB protein	YSEFLTFR	531,766374	2	812,430115	1	y6	E	PSAA | Photosystem I, PsaA/PsaB protein	P56766
						683,387522	1	y5	F	PSAA | Photosystem I, PsaA/PsaB protein	P56766
						423,235044	1	y3	T	PSAA | Photosystem I, PsaA/PsaB protein	P56766
ATCG00350.1	P56766	PSAA | Photosystem I, PsaA/PsaB protein	ALSIIQGR	429,263802	2	673,399149	1	y6	S	PSAA | Photosystem I, PsaA/PsaB protein	P56766
						586,367121	1	y5	I	PSAA | Photosystem I, PsaA/PsaB protein	P56766
						473,283057	1	y4	I	PSAA | Photosystem I, PsaA/PsaB protein	P56766
						360,198993	1	y3	Q	PSAA | Photosystem I, PsaA/PsaB protein	P56766
ATCG00340.1	P56767	PSAB | Photosystem I, PsaA/PsaB protein	QILIEPIFAQWIQSAHGK	1040,072924	2	1482,785209	1	y13	P	PSAB | Photosystem I, PsaA/PsaB protein	P56767
						1272,648381	1	y11	F	PSAB | Photosystem I, PsaA/PsaB protein	P56767
						499,262322	1	y5	S	PSAB | Photosystem I, PsaA/PsaB protein	P56767
						242,149918	1	b2	I	PSAB | Photosystem I, PsaA/PsaB protein	P56767
						355,233982	1	b3	L	PSAB | Photosystem I, PsaA/PsaB protein	P56767
ATCG00340.1	P56767	PSAB | Photosystem I, PsaA/PsaB protein	QILIEPIFAQWIQSAHGK	693,717708	3	1482,785209	1	y13	P	PSAB | Photosystem I, PsaA/PsaB protein	P56767
						1272,648381	1	y11	F	PSAB | Photosystem I, PsaA/PsaB protein	P56767
						926,484276	1	y8	W	PSAB | Photosystem I, PsaA/PsaB protein	P56767
						740,404963	1	y7	I	PSAB | Photosystem I, PsaA/PsaB protein	P56767
						499,262322	1	y5	S	PSAB | Photosystem I, PsaA/PsaB protein	P56767
						242,149918	1	b2	I	PSAB | Photosystem I, PsaA/PsaB protein	P56767
ATCG00340.1	P56767	PSAB | Photosystem I, PsaA/PsaB protein	TSYGFDVLLSSTSGPAFNAGR	1074,023825	2	1264,62804	1	y13	L	PSAB | Photosystem I, PsaA/PsaB protein	P56767
						1151,543976	1	y12	S	PSAB | Photosystem I, PsaA/PsaB protein	P56767
						1064,511947	1	y11	S	PSAB | Photosystem I, PsaA/PsaB protein	P56767
						789,400212	1	y8	G	PSAB | Photosystem I, PsaA/PsaB protein	P56767

						732,378748	1	y7	P	PSAB | Photosystem I, PsaA/PsaB protein	P56767
ATCG00340.1	P56767	PSAB | Photosystem I, PsaA/PsaB protein	TSYGFDVLLSSTSGPAFNAGR	716,351642	3	1151,543976	1	y12	S	PSAB | Photosystem I, PsaA/PsaB protein	P56767
						876,432241	1	y9	S	PSAB | Photosystem I, PsaA/PsaB protein	P56767
						789,400212	1	y8	G	PSAB | Photosystem I, PsaA/PsaB protein	P56767
						732,378748	1	y7	P	PSAB | Photosystem I, PsaA/PsaB protein	P56767
						564,288871	1	y5	F	PSAB | Photosystem I, PsaA/PsaB protein	P56767
ATCG00340.1	P56767	PSAB | Photosystem I, PsaA/PsaB protein	TPLANLIR	449,279452	2	699,451185	1	y6	L	PSAB | Photosystem I, PsaA/PsaB protein	P56767
						586,367121	1	y5	A	PSAB | Photosystem I, PsaA/PsaB protein	P56767
						515,330007	1	y4	N	PSAB | Photosystem I, PsaA/PsaB protein	P56767
						398,755612	2	y7	P	PSAB | Photosystem I, PsaA/PsaB protein	P56767
AT1G68830.1	Q9S713	STN7 | STT7 homolog STN7	LGEGSFGVVYK	578,305863	2	1042,520387	1	y10	G	STN7 | STT7 homolog STN7	Q9S713
						856,45633	1	y8	G	STN7 | STT7 homolog STN7	Q9S713
						409,244546	1	y3	V	STN7 | STT7 homolog STN7	Q9S713
						310,176132	1	y2	Y	STN7 | STT7 homolog STN7	Q9S713
						171,112804	1	b2	G	STN7 | STT7 homolog STN7	Q9S713
AT1G68830.1	Q9S713	STN7 | STT7 homolog STN7	SDSNLIQFNR	597,299101	2	790,456999	1	y6	L	STN7 | STT7 homolog STN7	Q9S713
						677,372935	1	y5	I	STN7 | STT7 homolog STN7	Q9S713
						564,288871	1	y4	Q	STN7 | STT7 homolog STN7	Q9S713
						203,066248	1	b2	D	STN7 | STT7 homolog STN7	Q9S713
AT1G68830.1	Q9S713	STN7 | STT7 homolog STN7	DGGFTETQLQELR	747,365169	2	1117,584778	1	y9	T	STN7 | STT7 homolog STN7	Q9S713
						887,494506	1	y7	T	STN7 | STT7 homolog STN7	Q9S713
						545,304186	1	y4	Q	STN7 | STT7 homolog STN7	Q9S713
						230,077147	1	b3	G	STN7 | STT7 homolog STN7	Q9S713
AT5G01920.1	Q9LZV4	STN8 | Protein kinase superfamily protein	NSTLVDDVR	509,76182	2	817,441408	1	y7	T	STN8 | Protein kinase superfamily protein	Q9LZV4
						716,39373	1	y6	L	STN8 | Protein kinase superfamily protein	Q9LZV4
						603,309666	1	y5	V	STN8 | Protein kinase superfamily protein	Q9LZV4
						274,187366	1	y2	V	STN8 | Protein kinase superfamily protein	Q9LZV4
AT5G01920.1	Q9LZV4	STN8 | Protein kinase superfamily protein	LIDFGAAADLR	581,316763	2	935,458121	1	y9	D	STN8 | Protein kinase superfamily protein	Q9LZV4
						820,431178	1	y8	F	STN8 | Protein kinase superfamily protein	Q9LZV4
						673,362764	1	y7	G	STN8 | Protein kinase superfamily protein	Q9LZV4
						545,304186	1	y5	A	STN8 | Protein kinase superfamily protein	Q9LZV4
AT5G01920.1	Q9LZV4	STN8 | Protein kinase superfamily protein	TRPDLSILDLDSGR	519,945888	3	662,310394	1	y6	D	STN8 | Protein kinase superfamily protein	Q9LZV4
						547,283451	1	y5	L	STN8 | Protein kinase superfamily protein	Q9LZV4
						319,172444	1	y3	S	STN8 | Protein kinase superfamily protein	Q9LZV4
						670,351865	1	b6	S	STN8 | Protein kinase superfamily protein	Q9LZV4
AT4G27800.1	P49599	TAP38, PPH1 | thylakoid-associated phosphatase 38	AFESVDR	412,200867	2	605,28893	1	y5	E	TAP38, PPH1 | thylakoid-associated phosphatase 38	P49599
						476,246337	1	y4	S	TAP38, PPH1 | thylakoid-associated phosphatase 38	P49599
						219,112804	1	b2	F	TAP38, PPH1 | thylakoid-associated phosphatase 38	P49599
AT4G27800.1	P49599	TAP38, PPH1 | thylakoid-associated phosphatase 38	SSDVVSYVR	506,258913	2	623,351137	1	y5	V	TAP38, PPH1 | thylakoid-associated phosphatase 38	P49599
						524,282723	1	y4	S	TAP38, PPH1 | thylakoid-associated phosphatase 38	P49599
						290,098276	1	b3	D	TAP38, PPH1 | thylakoid-associated phosphatase 38	P49599
AT4G27800.1	P49599	TAP38, PPH1 | thylakoid-associated phosphatase 38	SQDNISIIIADLGR	757,912287	2	957,572757	1	y9	S	TAP38, PPH1 | thylakoid-associated phosphatase 38	P49599
						644,3726	1	y6	I	TAP38, PPH1 | thylakoid-associated phosphatase 38	P49599
						531,288536	1	y5	A	TAP38, PPH1 | thylakoid-associated phosphatase 38	P49599
						345,22448	1	y3	L	TAP38, PPH1 | thylakoid-associated phosphatase 38	P49599
AT1G71500.1	Q9C9I7	PSB33 | Rieske (2Fe-2S) domain-containing protein	VLTPALR	385,250163	2	670,424636	1	y6	L	PSB33 | Rieske (2Fe-2S) domain-containing protein	Q9C9I7
						557,340572	1	y5	T	PSB33 | Rieske (2Fe-2S) domain-containing protein	Q9C9I7
						456,292893	1	y4	P	PSB33 | Rieske (2Fe-2S) domain-containing protein	Q9C9I7
						359,24013	1	y3	A	PSB33 | Rieske (2Fe-2S) domain-containing protein	Q9C9I7
						228,650085	2	y4	P	PSB33 | Rieske (2Fe-2S) domain-containing protein	Q9C9I7
AT1G71500.1	Q9C9I7	PSB33 | Rieske (2Fe-2S) domain-containing protein	TEAAAEIVFSGK	611,819335	2	992,541122	1	y10	A	PSB33 | Rieske (2Fe-2S) domain-containing protein	Q9C9I7
						850,466895	1	y8	A	PSB33 | Rieske (2Fe-2S) domain-containing protein	Q9C9I7
						231,097548	1	b2	E	PSB33 | Rieske (2Fe-2S) domain-containing protein	Q9C9I7
						302,134661	1	b3	A	PSB33 | Rieske (2Fe-2S) domain-containing protein	Q9C9I7
AT1G71500.1	Q9C9I7	PSB33 | Rieske (2Fe-2S) domain-containing protein	AQPGLTATNVNVDEVR	842,436657	2	1217,612056	1	y11	T	PSB33 | Rieske (2Fe-2S) domain-containing protein	Q9C9I7
						1045,527263	1	y9	T	PSB33 | Rieske (2Fe-2S) domain-containing protein	Q9C9I7
						731,368243	1	y6	N	PSB33 | Rieske (2Fe-2S) domain-containing protein	Q9C9I7
						742,888812	2	y14	P	PSB33 | Rieske (2Fe-2S) domain-containing protein	Q9C9I7
AT1G71500.1	Q9C9I7	PSB33 | Rieske (2Fe-2S) domain-containing protein	AQPGLTATNVNVDEVR	561,960197	3	731,368243	1	y6	N	PSB33 | Rieske (2Fe-2S) domain-containing protein	Q9C9I7
						518,256902	1	y4	D	PSB33 | Rieske (2Fe-2S) domain-containing protein	Q9C9I7
						403,229959	1	y3	E	PSB33 | Rieske (2Fe-2S) domain-containing protein	Q9C9I7
						200,102967	1	b2	Q	PSB33 | Rieske (2Fe-2S) domain-containing protein	Q9C9I7
AT1G29910.1	Q8VZ87	CAB3, AB180, LHCB1.2 | chlorophyll A/B binding protein 3	TVAKPK	322,210506	2	542,366058	1	y5	V	CAB3, AB180, LHCB1.2 | chlorophyll A/B binding protein 3	Q8VZ87
						443,297644	1	y4	A	CAB3, AB180, LHCB1.2 | chlorophyll A/B binding protein 3	Q8VZ87
						244,165568	1	y2	P	CAB3, AB180, LHCB1.2 | chlorophyll A/B binding protein 3	Q8VZ87
						201,123368	1	b2	V	CAB3, AB180, LHCB1.2 | chlorophyll A/B binding protein 3	Q8VZ87
						101,065322	2	b2	V	CAB3, AB180, LHCB1.2 | chlorophyll A/B binding protein 3	Q8VZ87
						200,631361	2	b4	K	CAB3, AB180, LHCB1.2 | chlorophyll A/B binding protein 3	Q8VZ87
AT1G29910.1	Q8VZ87	CAB3, AB180, LHCB1.2 | chlorophyll A/B binding protein 3	T[+80]VAKPK	362,193672	2	542,366058	1	y5	V	CAB3, AB180, LHCB1.2 | chlorophyll A/B binding protein 3	Q8VZ87
						443,297644	1	y4	A	CAB3, AB180, LHCB1.2 | chlorophyll A/B binding protein 3	Q8VZ87
						372,260531	1	y3	K	CAB3, AB180, LHCB1.2 | chlorophyll A/B binding protein 3	Q8VZ87
						244,165568	1	y2	P	CAB3, AB180, LHCB1.2 | chlorophyll A/B binding protein 3	Q8VZ87
						271,686667	2	y5	V	CAB3, AB180, LHCB1.2 | chlorophyll A/B binding protein 3	Q8VZ87
						183,112803	1	b2 -98	V	CAB3, AB180, LHCB1.2 | chlorophyll A/B binding protein 3	Q8VZ87
						254,149917	1	b3 -98	A	CAB3, AB180, LHCB1.2 | chlorophyll A/B binding protein 3	Q8VZ87
						382,24488	1	b4 -98	K	CAB3, AB180, LHCB1.2 | chlorophyll A/B binding protein 3	Q8VZ87
						127,578597	2	b3 -98	A	CAB3, AB180, LHCB1.2 | chlorophyll A/B binding protein 3	Q8VZ87
AT1G29910.1	Q8VZ87	CAB3, AB180, LHCB1.2 | chlorophyll A/B binding protein 3	GPSGSPWYGSDR	633,280908	2	1111,480313	1	y10	S	CAB3, AB180, LHCB1.2 | chlorophyll A/B binding protein 3	Q8VZ87
						880,394792	1	y7	P	CAB3, AB180, LHCB1.2 | chlorophyll A/B binding protein 3	Q8VZ87
						434,199387	1	y4	G	CAB3, AB180, LHCB1.2 | chlorophyll A/B binding protein 3	Q8VZ87
						155,081504	1	b2	P	CAB3, AB180, LHCB1.2 | chlorophyll A/B binding protein 3	Q8VZ87
AT1G29910.1	Q8VZ87	CAB3, AB180, LHCB1.2 | chlorophyll A/B binding protein 3	NRELEVIHSR	418,23007	3	512,293956	1	y4	I	CAB3, AB180, LHCB1.2 | chlorophyll A/B binding protein 3	Q8VZ87
						399,209892	1	y3	H	CAB3, AB180, LHCB1.2 | chlorophyll A/B binding protein 3	Q8VZ87
						642,320565	1	b5	E	CAB3, AB180, LHCB1.2 | chlorophyll A/B binding protein 3	Q8VZ87

						741,388979	1	b6	V	CAB3, AB180, LHCB1.2 | chlorophyll A/B binding protein 3	Q8VZ87
AT1G29910.1	Q8VZ87	CAB3, AB180, LHCB1.2 | chlorophyll A/B binding protein 3	ELEVIHSR	491,769448	2	740,404963	1	y6	E	CAB3, AB180, LHCB1.2 | chlorophyll A/B binding protein 3	Q8VZ87
						611,36237	1	y5	V	CAB3, AB180, LHCB1.2 | chlorophyll A/B binding protein 3	Q8VZ87
						512,293956	1	y4	I	CAB3, AB180, LHCB1.2 | chlorophyll A/B binding protein 3	Q8VZ87
						399,209892	1	y3	H	CAB3, AB180, LHCB1.2 | chlorophyll A/B binding protein 3	Q8VZ87
						243,133933	1	b2	L	CAB3, AB180, LHCB1.2 | chlorophyll A/B binding protein 3	Q8VZ87
AT1G29910.1	Q8VZ87	CAB3, AB180, LHCB1.2 | chlorophyll A/B binding protein 3	ELEVIHSR	328,182057	3	512,293956	1	y4	I	CAB3, AB180, LHCB1.2 | chlorophyll A/B binding protein 3	Q8VZ87
						399,209892	1	y3	H	CAB3, AB180, LHCB1.2 | chlorophyll A/B binding protein 3	Q8VZ87
						306,184823	2	y5	V	CAB3, AB180, LHCB1.2 | chlorophyll A/B binding protein 3	Q8VZ87
						256,650616	2	y4	I	CAB3, AB180, LHCB1.2 | chlorophyll A/B binding protein 3	Q8VZ87
						200,108584	2	y3	H	CAB3, AB180, LHCB1.2 | chlorophyll A/B binding protein 3	Q8VZ87
AT1G29910.1	Q8VZ87	CAB3, AB180, LHCB1.2 | chlorophyll A/B binding protein 3	ELEVIHS[+80]RWAMLGALGC[+57]VFPELLAR	983,162257	3	1161,608491	1	y10	G	CAB3, AB180, LHCB1.2 | chlorophyll A/B binding protein 3	Q8VZ87
						845,487965	1	y7	F	CAB3, AB180, LHCB1.2 | chlorophyll A/B binding protein 3	Q8VZ87
						698,419551	1	y6	P	CAB3, AB180, LHCB1.2 | chlorophyll A/B binding protein 3	Q8VZ87
						372,176526	1	b3	E	CAB3, AB180, LHCB1.2 | chlorophyll A/B binding protein 3	Q8VZ87
						471,24494	1	b4	V	CAB3, AB180, LHCB1.2 | chlorophyll A/B binding protein 3	Q8VZ87
AT1G29910.1	Q8VZ87	CAB3, AB180, LHCB1.2 | chlorophyll A/B binding protein 3	NGVKFGEAVWFK	691,366787	2	983,498529	1	y8	F	CAB3, AB180, LHCB1.2 | chlorophyll A/B binding protein 3	Q8VZ87
						294,181218	1	y2	F	CAB3, AB180, LHCB1.2 | chlorophyll A/B binding protein 3	Q8VZ87
						172,071667	1	b2	G	CAB3, AB180, LHCB1.2 | chlorophyll A/B binding protein 3	Q8VZ87
						271,140081	1	b3	V	CAB3, AB180, LHCB1.2 | chlorophyll A/B binding protein 3	Q8VZ87
AT1G29910.1	Q8VZ87	CAB3, AB180, LHCB1.2 | chlorophyll A/B binding protein 3	NGVKFGEAVWFK	461,24695	3	650,366058	1	y5	A	CAB3, AB180, LHCB1.2 | chlorophyll A/B binding protein 3	Q8VZ87
						480,260531	1	y3	W	CAB3, AB180, LHCB1.2 | chlorophyll A/B binding protein 3	Q8VZ87
						294,181218	1	y2	F	CAB3, AB180, LHCB1.2 | chlorophyll A/B binding protein 3	Q8VZ87
AT1G29910.1	Q8VZ87	CAB3, AB180, LHCB1.2 | chlorophyll A/B binding protein 3	FGEAVWFK	492,252903	2	836,430115	1	y7	G	CAB3, AB180, LHCB1.2 | chlorophyll A/B binding protein 3	Q8VZ87
						650,366058	1	y5	A	CAB3, AB180, LHCB1.2 | chlorophyll A/B binding protein 3	Q8VZ87
						480,260531	1	y3	W	CAB3, AB180, LHCB1.2 | chlorophyll A/B binding protein 3	Q8VZ87
						205,097154	1	b2	G	CAB3, AB180, LHCB1.2 | chlorophyll A/B binding protein 3	Q8VZ87
AT2G05100.1	Q9SHR7	LHCB2.1, LHCB2 | photosystem II light harvesting complex gene 2.1	STPQSIWYGPDRPK	816,412454	2	1018,510491	1	y8	W	LHCB2.1, LHCB2 | photosystem II light harvesting complex gene 2.1	Q9SHR7
						832,431178	1	y7	Y	LHCB2.1, LHCB2 | photosystem II light harvesting complex gene 2.1	Q9SHR7
						669,367849	1	y6	G	LHCB2.1, LHCB2 | photosystem II light harvesting complex gene 2.1	Q9SHR7
						722,3726	2	y12	P	LHCB2.1, LHCB2 | photosystem II light harvesting complex gene 2.1	Q9SHR7
AT2G05100.1	Q9SHR7	LHCB2.1, LHCB2 | photosystem II light harvesting complex gene 2.1	STPQSIWYGPDRPK	544,610728	3	832,431178	1	y7	Y	LHCB2.1, LHCB2 | photosystem II light harvesting complex gene 2.1	Q9SHR7
						669,367849	1	y6	G	LHCB2.1, LHCB2 | photosystem II light harvesting complex gene 2.1	Q9SHR7
						612,346386	1	y5	P	LHCB2.1, LHCB2 | photosystem II light harvesting complex gene 2.1	Q9SHR7
						189,086983	1	b2	T	LHCB2.1, LHCB2 | photosystem II light harvesting complex gene 2.1	Q9SHR7
AT2G05100.1	Q9SHR7	LHCB2.1, LHCB2 | photosystem II light harvesting complex gene 2.1	ELEVIHS[+80]RWAMLGALGC[+57]TFPEILSK	979,818267	3	686,408317	1	y6	P	LHCB2.1, LHCB2 | photosystem II light harvesting complex gene 2.1	Q9SHR7
						347,228896	1	y3	L	LHCB2.1, LHCB2 | photosystem II light harvesting complex gene 2.1	Q9SHR7
						234,144832	1	y2	S	LHCB2.1, LHCB2 | photosystem II light harvesting complex gene 2.1	Q9SHR7
						1185,116483	2	y21 -98	I	LHCB2.1, LHCB2 | photosystem II light harvesting complex gene 2.1	Q9SHR7
						566,79854	2	b9 -98	W	LHCB2.1, LHCB2 | photosystem II light harvesting complex gene 2.1	Q9SHR7
ATCG00540.1	P56771	PETA | photosynthetic electron transfer A	YPIYVGGNR	519,771991	2	778,420613	1	y7	I	PETA | photosynthetic electron transfer A	P56771
						665,336549	1	y6	Y	PETA | photosynthetic electron transfer A	P56771
						502,273221	1	y5	V	PETA | photosynthetic electron transfer A	P56771
						403,204807	1	y4	G	PETA | photosynthetic electron transfer A	P56771
ATCG00540.1	P56771	PETA | photosynthetic electron transfer A	EVIDIIPR	477,784567	2	726,450851	1	y6	I	PETA | photosynthetic electron transfer A	P56771
						613,366787	1	y5	D	PETA | photosynthetic electron transfer A	P56771
						498,339844	1	y4	I	PETA | photosynthetic electron transfer A	P56771
						385,25578	1	y3	I	PETA | photosynthetic electron transfer A	P56771
						229,118283	1	b2	V	PETA | photosynthetic electron transfer A	P56771
ATCG00540.1	P56771	PETA | photosynthetic electron transfer A	GLELLVSEGESIK	687,379757	2	848,435989	1	y8	V	PETA | photosynthetic electron transfer A	P56771
						749,367575	1	y7	S	PETA | photosynthetic electron transfer A	P56771
						300,155397	1	b3	E	PETA | photosynthetic electron transfer A	P56771
						413,239461	1	b4	L	PETA | photosynthetic electron transfer A	P56771
ATCG00540.1	P56771	PETA | photosynthetic electron transfer A	GLELLVSEGESIK	458,58893	3	749,367575	1	y7	S	PETA | photosynthetic electron transfer A	P56771
						533,292953	1	y5	G	PETA | photosynthetic electron transfer A	P56771
						300,155397	1	b3	E	PETA | photosynthetic electron transfer A	P56771
						413,239461	1	b4	L	PETA | photosynthetic electron transfer A	P56771
ATCG00720.1	P56773	PETB | photosynthetic electron transfer B	VYLTGGFK	442,747453	2	785,419216	1	y7	Y	PETB | photosynthetic electron transfer B	P56773
						622,355888	1	y6	L	PETB | photosynthetic electron transfer B	P56773
						509,271824	1	y5	T	PETB | photosynthetic electron transfer B	P56773
						408,224145	1	y4	G	PETB | photosynthetic electron transfer B	P56773
ATCG00720.1	P56773	PETB | photosynthetic electron transfer B	IVTGVPDAIPVIGSPLVELLR	1079,645922	2	1292,793649	1	y12	P	PETB | photosynthetic electron transfer B	P56773
						983,588407	1	y9	G	PETB | photosynthetic electron transfer B	P56773
						371,228896	1	b4	G	PETB | photosynthetic electron transfer B	P56773
						470,29731	1	b5	V	PETB | photosynthetic electron transfer B	P56773
						284,178675	2	b6	P	PETB | photosynthetic electron transfer B	P56773
ATCG00720.1	P56773	PETB | photosynthetic electron transfer B	IVTGVPDAIPVIGSPLVELLR	720,099707	3	1292,793649	1	y12	P	PETB | photosynthetic electron transfer B	P56773
						1096,672471	1	y10	I	PETB | photosynthetic electron transfer B	P56773
						983,588407	1	y9	G	PETB | photosynthetic electron transfer B	P56773
						839,534915	1	y7	P	PETB | photosynthetic electron transfer B	P56773
						284,178675	2	b6	P	PETB | photosynthetic electron transfer B	P56773
ATCG00720.1	P56773	PETB | photosynthetic electron transfer B	GSASVGQSTLTR	582,304383	2	861,478856	1	y8	V	PETB | photosynthetic electron transfer B	P56773
						762,410442	1	y7	G	PETB | photosynthetic electron transfer B	P56773
						705,388979	1	y6	Q	PETB | photosynthetic electron transfer B	P56773
						577,330401	1	y5	S	PETB | photosynthetic electron transfer B	P56773

:ession
